# Relevance of intra-hospital patient movements for the spread of healthcare-associated infections within hospitals - a mathematical modeling study

**DOI:** 10.1371/journal.pcbi.1008600

**Published:** 2021-02-03

**Authors:** Hannan Tahir, Luis Eduardo López-Cortés, Axel Kola, Dafna Yahav, André Karch, Hanjue Xia, Johannes Horn, Konrad Sakowski, Monika J. Piotrowska, Leonard Leibovici, Rafael T. Mikolajczyk, Mirjam E. Kretzschmar

**Affiliations:** 1 Julius Center for Health Sciences & Primary Care, University Medical Center Utrecht, Utrecht University, Utrecht, The Netherlands; 2 Unidad Clínica de Enfermedades Infecciosas, Microbiología y Medicina Preventiva, Hospital Universitario Virgen Macarena, Sevilla, Spain; 3 Institute of Institute of Hygiene and Environmental Medicine, Charité- University Medicine Berlin, Berlin, Germany; 4 Infectious Diseases Unit, Rabin Medical Center, Beilinson Hospital, Petah-Tiqva, Israel; 5 Institute for Epidemiology and Social Medicine, University of Münster, Münster, Germany; 6 Institute for Medical Epidemiology, Biometry, and Informatics (IMEBI), Interdisciplinary Center for Health Sciences, Medical Faculty of the Martin Luther University Halle-Wittenberg, Halle, Germany; 7 Institute of Applied Mathematics and Mechanics, University of Warsaw, Warsaw, Poland; 8 Institute of High Pressure Physics, Polish Academy of Sciences, Warsaw, Poland; 9 Department of Medicine E; Rabin Medical Center, Beilinson Hospital, Petah-Tiqva, Israel; University of Zurich, SWITZERLAND

## Abstract

The aim of this study is to analyze patient movement patterns between hospital departments to derive the underlying intra-hospital movement network, and to assess if movement patterns differ between patients at high or low risk of colonization. For that purpose, we analyzed patient electronic medical record data from five hospitals to extract information on risk stratification and patient intra-hospital movements. Movement patterns were visualized as networks, and network centrality measures were calculated. Next, using an agent-based model where agents represent patients and intra-hospital patient movements were explicitly modeled, we simulated the spread of multidrug resistant enterobacteriacae (MDR-E) inside a hospital. Risk stratification of patients according to certain ICD-10 codes revealed that length of stay, patient age, and mean number of movements per admission were higher in the high-risk groups. Movement networks in all hospitals displayed a high variability among departments concerning their network centrality and connectedness with a few highly connected departments and many weakly connected peripheral departments. Simulating the spread of a pathogen in one hospital network showed positive correlation between department prevalence and network centrality measures. This study highlights the importance of intra-hospital patient movements and their possible impact on pathogen spread. Targeting interventions to departments of higher (weighted) degree may help to control the spread of MDR-E. Moreover, when the colonization status of patients coming from different departments is unknown, a ranking system based on department centralities may be used to design more effective interventions that mitigate pathogen spread.

## Author summary

Pathogens including multidrug resistant enterobacteriacae (MDR-E) inside hospital settings are associated with higher morbidity, mortality, and healthcare costs. Better understanding of the transmission routes of these pathogens is required to develop targeted and efficient measures to contain the spread of MDR-E in a hospital. We analyzed datasets from five hospitals in different countries to explore patient movement patterns between departments of these hospitals (intra-hospital movements). We assessed whether movement patterns differ between patients at high or low risk of colonization. Our results show that in every intra-hospital network, there exist a few departments which are strongly connected and many peripheral departments which are loosely connected. High-risk patients stay on average longer in the hospital, and move more frequently between departments than low-risk patients. Targeting interventions to strongly connected departments may help to reduce pathogen spread inside the hospital. To explore this, we simulated the spread of MDR-E inside one hospital using an agent-based model that includes patient movements. In the simulations, we found positive correlations between departments’ prevalence and network characteristics such as degree and weighted degree, thus highlighting the importance of targeting interventions to departments of higher (weighted) degree to control the spread of MDR-E.

## Introduction

Multidrug resistant enterobacteriacae (MDR-E) are a common cause of hospital-acquired infections (HAIs) [1–4] and are considered a major public health threat. HAIs due to MDR-E are associated with higher morbidity, mortality, and healthcare costs [5,6]. A better understanding of the transmission routes of MDR-E pathogens may provide valuable insight to develop more effective and targeted infection control measures. When dealing with the spread of MDR-E in a single hospital, several factors such as contact precautions, inadequate hygiene protocols, and prolonged hospital stays play an important role. However, in recent years, inter-hospital patient movements between healthcare facilities have been recognized as an important route of transmission of pathogens between healthcare facilities. Various studies have used data on inter-hospital transfers of patients to construct healthcare networks. Based on those networks, various innovative infection control measures were proposed to contain the spread of HAIs [7–10]. Moreover, the burden of HAIs in a healthcare system has been proposed to be dependent on the structure of the inter-hospital network [9–12].

Similar to the role of inter-hospital patient movements, patient movements between the departments of a single hospital (intra-hospital movements) may contribute to spread of pathogens within a hospital. The effect of intra-hospital movements of patients on pathogen spread in a single hospital, however, is not fully understood. Various studies have reported the spread of MDR-E pathogens inside a hospital [13–17]. However, such studies were either limited to intensive care units (ICUs), or to some specific medical specialty departments, and patient movements between the departments were not assessed. Few modeling studies related to the spread of Methicillin-resistant *Staphylococcus aureus* (MRSA) [18,19] and Carbapenem-resistant *Klebsiella pneumoniae* [20] have evaluated the role of patient movements inside a hospital, but either very few departments were considered or departments other than ICUs were considered equal in terms of department structures (e.g. similar number of beds per department, similar movement rates between the departments etc.). These studies did not account for the hospital department network when investigating the role of intra-hospital patient movements. Certain departments such as ICUs, emergency and surgery departments tend to have more patient movements than others. Applying infection control strategies to those departments may, therefore, help to limit the spread of the pathogen. A detailed understanding of how patient movements inside a hospital contribute to the spread and prevalence of pathogens in departments and in the entire hospital may help to develop more effective infection control strategies.

Several risk factors for acquiring HAIs due to MDR-E pathogens have been reported in the literature such as prolonged ICU or hospital stays [21,22], prior antibiotic usage [21,23–25], older age [26], renal dysfunction [26,27], mechanical ventilation [27], and recent invasive surgical procedures [22,25]. However, a patient’s risk of acquiring colonization varies between patients, and not all patients are equally likely to become colonized with MDR-E pathogens. Multiple illnesses such as cancer, diabetes mellitus, dialysis, chronic renal disease, chronic alcoholism, chronic liver disease, and solid-organ transplantation have also been identified as risk factors for infection with MDR-E pathogens, as they weaken host defenses and thus increase host susceptibility to developing an infection [27–31]. It is, however, not yet clear whether such illnesses are associated with acquisition and colonization, or only with infection [27]. It is also worth noting here that a patient’s susceptibility to acquiring colonization may not differ between patients with or without chronic diseases, but that observed differences in colonization rates between patients might be due to difference in exposure. Patients with severe disorders or chronic diseases are more likely to be in need of repeated hospital admission, and require more intense care from healthcare workers. Frequent contacts with healthcare workers may put such patients at high risk for acquiring colonization during their hospital stay.

The aim of this study is to assess and understand patient movement patterns in hospitals from different countries based on electronic hospital information systems data. Our analysis includes a stratification of movement patterns by risk level based on ICD-10 codes at discharge. To study the impact of intra-hospital patient movements on pathogen spread, we performed simulations using an agent-based transmission model including patient movements between departments. We analyzed the association between departments’ prevalence and various network centrality measures obtained from the agent-based simulations. Finally, we discuss implications for targeted intervention measures to reduce pathogen spread in hospitals.

## Material and methods

### Data

We obtained routine hospital admission data of five hospitals from Spain, The Netherlands, Germany, and Israel. The pseudonymized admission data were extracted from electronic hospital information systems and do not include any sensitive information. Participating hospitals were: University Medical Center Utrecht (UMCU), Utrecht, The Netherlands; Hospital Universitario Virgen Macarena (HUVM), Seville, Spain; Charité Universitätsmedizin (CUM), Berlin, Germany; Beilinson Hospital (BH), Rabin Medical Center, Petah Tikva, Israel; and Universitätsklinikum Halle (UKH), Halle, Germany. Basic details of every dataset are given in *Table 1*.

**Table 1:** Descriptive data of participating hospitals.

Hospitals	University Medical Center Utrecht, The Netherlands (UMCU)	Hospital Universitario Virgen Macarena, Spain (HUVM)	Charité Universitäts-medizin, Germany (CUM)	Beilinson Hospital, Israel (BH)	Universitäts klinikum Halle, Germany (UKH)
Data Period	01.01.2014–31.12.2017	01.01.2016–30.01.2017	01.01.2016–31.12.2016	01.01.2012–31.12.2017	01.01.2017–31.12.2017
Hospital Size (number of beds)	1042	950	3011	800	950
Number of Departments	61	36	58	26	58
Number of patients per year *	30,823	26,724	416,751	17,410	32,863
Number of admissions per year **	81,516	34,364	841,221	33,754	47,279
Total number of admissions (N) **	326,064	37,227	841,221	202,524	47,279
Number of admissions used in the analysis (N (%))	117,758 (36.12)	28,191 (75.73)	124,946 (14.85)	169,541 (83.71)	37,977 (80.33)

* A patient can be admitted more than once. Every patient gets a unique patient ID and it remains unchanged for future admissions.

** Admissions include both in-patient and out-patient admissions. For every (re)admission, a unique admission ID is assigned to a patient.

The provided data included: patient ID, hospital ID, patient birth year, admission and discharge dates, and ICD-10 diagnosis codes. Additionally, the names of all departments in which patients stayed during their hospital stay were provided for the respective time periods. Data extraction periods were different for every hospital. Since information on patients who were transferred to other hospitals was not available from every hospital, we considered those movements as discharges.

We have, further, excluded admissions with a hospital length of stay (LOS) of less than a day (outpatient admissions) and only considered inpatient admissions (hospital LOS > 1 day) in the study. The reason behind this exclusion is that outpatients may not be exposed to the parts of the hospital where inpatients stay, and therefore do not contribute to spread of pathogens. Moreover, admissions to psychiatric departments were excluded because patients in those departments tend to have completely different (often very long) LOS and are usually not bedridden. We also excluded admissions in obstetrics departments because we observed that newly born children do not immediately get their own patient and admission ID, but those of their mothers instead. Thus, it is difficult to differentiate between newborn children from their mothers in our data. Moreover, in obstetrics departments, transfers to other departments are rare and the duration of stay is short. *Table 1*reports the total number of admissions included in the current study from every hospital after the above exclusions.

### Patient risk stratification

Patients having severe disorders or chronic diseases and immunocompromised patients may have a higher risk of acquiring colonization and subsequent infection. A risk stratification into low-risk and high-risk patients [32], was implemented based on certain diagnoses (ICD10 codes), which are known to be associated with patient disabilities (*Table 2*). This risk stratification was applied consecutively to any further hospitalization of the patient, i.e. once a patient is defined as a high-risk person, the patient will automatically be defined as high-risk for any consecutive hospitalization independent of the respective diagnoses.

**Table 2:** Patient risk stratification. A patient having any of the mentioned ICD-10 codes was considered as a high-risk patient.

Disease	ICD-10 Code(s)
Cancer	C00-C96
Diabetes mellitus	E10-E14
Heart failure	I50
Chronic kidney disease (moderate or severe)	N18.3-N18.6
Immune system disease	D80-D89
Systemic sclerosis and other systemic involvement of connective tissue	M34-M35
Psoriasis (chronic skin disease)	L40
Abnormal immunological findings in serum	R76

### Intra-hospital movements

When a patient is transferred from one department to another, we counted this transfer as a single movement. From department-level data, we extracted such intra-hospital movements for every hospital stay. Patient movements within a single department were not considered in this study.

We did not put any constraints on a minimum time between movements. However, if a patient stays in another department only for a short time, the probability that transmission occurs is very small, so short stays do not contribute much to overall transmissions in the hospital. Using intra-hospital movements data, we created a directed movement network for each risk group and for each hospital in order to visualize patient movements patterns. In such a network, nodes represent departments and links represent patient transfers between the departments. We further computed network statistics such as degree, weighted degree, graph density, average path length, average clustering coefficient and network diameter.

### Data analysis tools

*Python Pandas* [33] was used for data cleaning, filtering, stratification, and analysis. From *Python Pandas*, patients’ intra-hospital movements for each hospital were exported as weighted edge-lists, where weights represent numbers of patient movements in each direction. These weighted edge-lists were later imported into *Gephi* software [34] for network visualization and computation of network statistics. In this study, we used the mean and standard deviation for most of the descriptive quantities, but for some other variables we used median and show interquartile range (IQR).

### Agent-based model

In order to evaluate the role of patient intra-hospital movements and their implications towards MDR-E spread, we developed a discrete-event agent-based model (ABM) to simulate the spread of a pathogen inside a hospital. To demonstrate our methods, we performed simulations for the Spanish hospital HUVM, for which we had the most complete data. The model was built using *Python* library *Mesa* which is an open source ABM framework [35]. In the HUVM hospital, there were 34 departments present in total. Number of beds in each department was estimated from the HUVM dataset using mean number of patients present every day in each department (*S10 Fig*).

In the model, we simulated patients explicitly as individual agents and every agent had several descriptive attributes, namely unique id, risk score, length of stay (LOS), disease state, department number, and bed number. In the ABM, agents’ attributes were updated in discrete time steps of 5 minutes. During the simulation, the new patient’s arrivals process is modeled as a Poisson process with estimated daily arrival rate. Moreover, for all the simulations, patients were uniformly distributed at admission to available beds in the departments. At the time of admission, a LOS in discrete time units was assigned to every individual agent from an exponential probability density function (estimated from the HUVM dataset). At every time step, patient’s LOS was decremented by 1, and once the LOS for a patient reached zero, the patient was discharged from the hospital. Our model does not account for changes in the patient population due to death.

In the data, for patients who moved at least twice during their stay in the HUVM hospital, we observed that in 81% of such movements, patients were moved back to the previous ward. Based on this observation, we implemented in the ABM that there is an 81% chance to return to the previous ward for patients with two or more movements. For the remaining 19% of the movements, patients follow the department selection algorithm through a preference matrix explained below. Daily patient movement rates to other departments were estimated for every department of the HUVM hospital (*S11 Fig*). These movement rates were then divided by the size of the departments to obtain department-specific daily movement probabilities. Given those daily movement probabilities, numbers of patients to be moved to other departments were calculated for every department every simulated day, and added to a department specific counter. This counter kept track of the number of patients to be moved from every department.

For each patient movement, we determined a future department using a preference matrix composed of preference probabilities [36]. For this preference matrix, a pivot table was first computed based on every department’s weighted in-degree and weighted out-degree from the HUVM hospital. This pivot table was then normalized row-wise to obtain preference probabilities. Given those preference probabilities [36], a patient’s next department was selected and the patient is moved to the new department. It is worthwhile to note, that there are fixed numbers of beds per department, which can all be occupied at a certain moment. If all beds in potential new departments were occupied at a certain moment, the patient stayed in the current location and the above explained procedure is repeated in the next time step.

Each patient also has a disease state: *susceptible*, *colonized*, or *infected*. A patient in a *susceptible* state can immediately become colonized after being exposed to MDR-E pathogens. A patient in a *colonized* state remains asymptomatically colonized with MDR-E and can transmit the pathogen to others upon contact. A patient in an *infected* state is symptomatically colonized showing disease symptoms. Infected patients can still spread the pathogen to others. In the model, we assumed that the transition from *susceptible* to *infected* requires passing through *colonized* and, therefore, neglect a direct pathway from *susceptible* to *infected*. More details on the disease progression can be found in *S12 Fig*. When a patient becomes infected in the model, no further movement of that patient will be allowed. This assumption is based on discussions with clinicians from the participating hospitals, where the majority of the patients after infection diagnosis are only moved to other departments in case of emergencies.

Intra-hospital models usually assume that transmission occurs via the cross-transmission route, which involves effective contacts between patients and healthcare workers (HCWs). Since we did not model HCWs explicitly, the transmission was implemented as a force of infection (FOI), which gives the probability per unit of time *t* for a susceptible patient to acquire the pathogen and to become colonized. The FOI was dependent on the transmission parameter *β*, the number of colonized and infected patients, and the total number of patients present in a given department. Martin et al. [27] reported that approximately 5% of the patients with gastrointestinal carriage of Carbapenem-resistant *Klebsiella pneumoniae* developed an infection. We calibrated our model to achieve a similar cumulative percentage of infected patients using a probability of 0.012/day in Bernoulli trials. Once the disease state of a patient was changed to *infected*, the LOS of that patient was increased by three days. After the end of this extended LOS period, the patient returned back to a *colonized* state. Since infected patients return back to a *colonized* state, they may become infected again in the model. The increase in the LOS by three days for infected patients was a parameter in our model; thus, we also checked the effects of varying this parameter on the department prevalence (*S13–S15 Figs*). In our model, daily transmission rates for both high-risk and low-risk patients were the same. However, it was assumed that there was a difference in the mean LOS, with a slightly longer LOS for high-risk patients when compared to the low-risk group. Further details on the model and parameters are given in *S1 and S1A Text,* respectively.

To evaluate the role of patient movements for pathogen spread, we tested the four different scenarios using the ABM described below. We apply these scenarios starting from day 30 so that a stable hospital population of susceptible patients can be assumed.

- *Scenario 1*: On day 30 (simulation time) one colonized patient was admitted to the highest weighted degree centrality department (ICU–department 8). The motivation for this scenario is to highlight the impact of patient movements on pathogen spread in case of a single imported case (no continuous inflow of colonized patients).
- *Scenario 2*: continuous inflow of 1% colonized patients from day 30 onwards. We used a probability of 0.01 for a patient to arrive in a colonized state into the hospital. These colonized patients were then randomly distributed to all departments of the hospital.
- *Scenario 3*: continuous inflow of 5% colonized patients from day 30 onwards, distributed randomly to all departments of the hospital.
- *Scenario 4*: continuous inflow of 15% colonized patients from day 30 onwards, distributed randomly to all departments of the hospital.

In all scenarios, we tested different values of the transmission parameter *β* (range 0.0005–0.30 per day). Spearman’s rank correlation coefficients between departments`network characteristics and departments`prevalence were computed to evaluate the association between these two quantities. For scenarios with continuous inflow of colonized patients, we used stable prevalence states for the Spearman’s rank correlation test.

## Results

### Descriptive statistics of hospital data

Descriptive statistics for each risk group for every hospital are shown in *Table 3*. In all hospitals, proportions of male patients and proportions of male admissions in the high-risk groups were always higher when compared to proportions of low-risk male patients and admissions. Proportions of high-risk admissions vary between 30.20%– 36.69% between the hospitals with CUM being the lowest and HUVM having the highest proportion.

**Table 3:** Descriptive statistics for data included in the analysis for participating hospitals.

Hospitals		UMCU		HUVM		CUM		BH		UKH	
		Low-risk	High-risk	Low-risk	High-risk	Low-risk	High-risk	Low-risk	High-risk	Low-risk	High-risk
Patients	Total (N)	52590	16870	15368	6910	68755	23008	70369	26480	19491	8487
	Males (%)	53.16	57.27	50.78	55.18	48.24	56.10	52.98	54.56	47.60	56.00
Admissions	Total (N (%))	75147 (63.82)	42611 (36.18)	17848 (63.31)	10343 (36.69)	87219 (69.80)	37727 (30.20)	109985 (64.87)	59556 (35.13)	24071 (63.38)	13906 (36.62)
	Males (%)	52.91	57.67	51.70	56.39	48.26	57.30	52.89	55.25	48.6	57.92
Admissions per day (SD)		51.43 (21.02)	29.16 (14.03)	44.49 (13.57)	25.67 (8.45)	238.3 (133.8)	103.07 (85.31)	50.17 (17.15)	27.16 (11.37)	65.94 (28.77)	38.09 (20.64)
Mean LOS (Days (SD))		5.52 (9.88)	7.19 (11.46)	6.09 (8.84)	8.49 (9.59)	5.31 (8.06)	8.74 (13.69)	4.85 (7.16)	6.61 (9.55)	5.52 (7.94)	10.09 (12.7)
Patient Age (Median (IQR))		38 (6–62)	64 (50–72)	57 (34–74)	72 (61–81)	52 (31–69)	66 (54–76)	65 (47–78)	72 (62–81)	52 (27–67)	67 (57–77)

**Fig 1:**
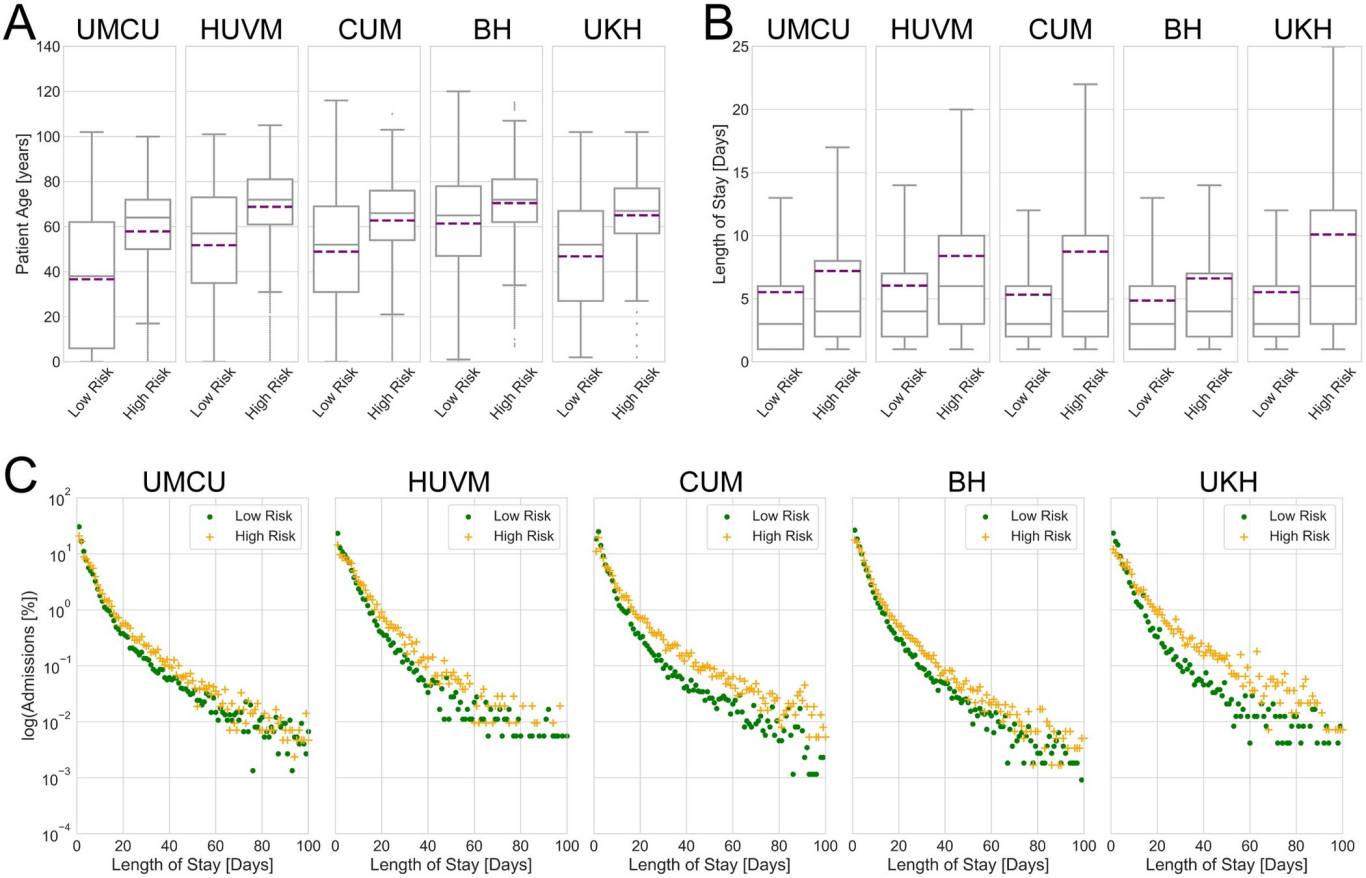
(A) Box plots showing patient age distribution for each hospital and risk group, (B) Box plots for admission LOS distribution for each hospital and risk group. In A and B, purple lines in the boxes show mean value whereas the grey line in the boxes show median of the data. (C) Proportion of admissions versus LOS on log-linear scale for each risk group in every hospital. To better visualize the trends between the risk groups at smaller LOS, proportion of admissions with LOS over 100 days are not shown in C. There are however, few data points above 100 days LOS (see S1 Numerical Data for complete data).

Patients in the high-risk groups were on average older than low-risk patients (*Fig 1A*). *Fig 1A* shows that BH hospital had the highest mean age in both risk groups. *Fig 1B* displays LOS distributions where we observed longer mean LOS in the high-risk groups when compared to low-risk groups in each hospital. High-risk groups in CUM, UKH and HUVM had large variability in the LOS as shown by the box plots (*Fig 1B*). We further plotted the proportion of admissions versus LOS in all the hospitals and risk groups on a log-linear scale to display the distributions of LOS. *Fig 1C* shows that in all hospitals, a large proportion of admissions in both risk groups had short LOS. Moreover, low-risk groups have a higher proportion of patients with short LOS (between 1–4 days). For both risk groups in each hospital, their LOS was approximately exponentially distributed.

### Patient movements

**Fig 2:**
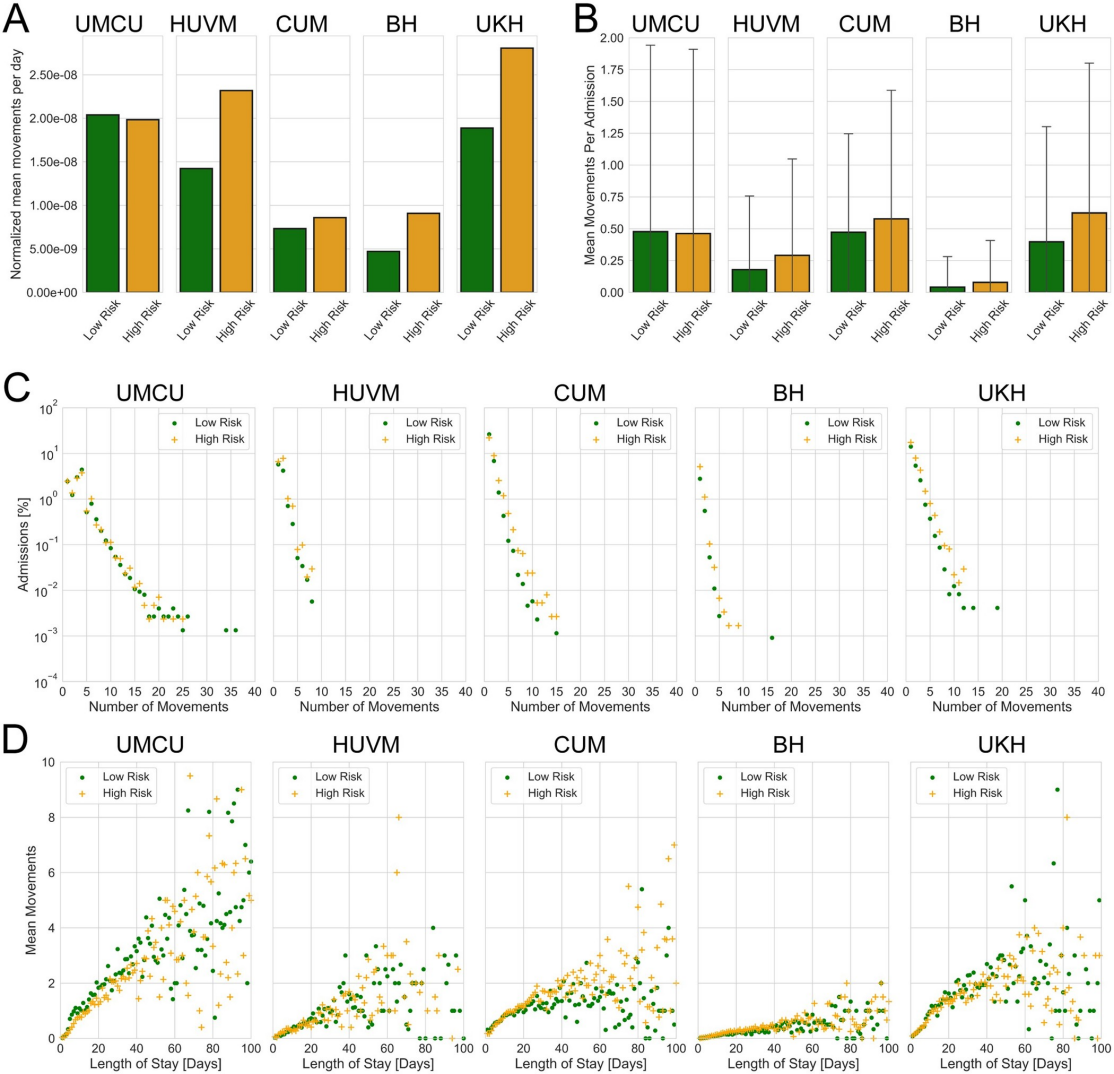
Patients movement results. (A) Mean number of movements per day normalized by hospital size, number of departments and the number of admissions per year in each risk group for every hospital. (B) Mean number of movements per hospital admission for each risk group and for each hospital. Error bars show standard deviation. (C) Proportion of admissions versus number of movements in each risk group for every hospital on log-linear scale. It is worth noting here that major proportion of admissions in each hospital has zero movements (UMCU (Low-risk 86.62%, High-risk 87.06%), HUVM (Low-risk 89.05%, High-risk 83.79%), CUM (Low-risk 64.81%, High-risk 64.43%), BH (Low-risk 96.6%, High-risk 93.63%), and UKH (Low-risk 76.45%, High-risk 66.01%)). (D) Mean number of movements versus admission LOS for each risk group and every hospital. To better visualize the trends between the risk groups at smaller LOS, data over 100 days LOS are not shown in D. There are however, few data points above 100 days LOS (see S2 Numerical Data for complete data).

Patient intra-hospital movements were estimated from patient transfers between the departments in every admission. *Fig 2A* shows mean movements per day normalized by hospital size, number of departments, and number of admissions per year in each risk group for every hospital. It is clear from *Fig 2A* that UKH had the highest mean intra-hospital movements per day in the high-risk group. Moreover, high-risk groups in all hospitals had a higher mean number of movements per day except for the UMCU. Mean number of intra-hospital movements per hospital admission are shown in *Fig 2B*. High-risk groups had notably higher means when compared to low-risk groups, except for the UMCU where there was little difference between the risk groups. BH had the lowest mean number of movements per admission.

The majority of patients in each hospital did not move between the departments during their hospital stay. For UMCU, HUVM and BH, more than 83% of admissions in both risk groups had no movements. This effect was even more pronounced in the BH where patients in 93% of the admissions in the high-risk group, and 96% in the low-risk group, did not move between the departments. The German hospitals CUM and UHK had smaller proportions of admissions with zero movements (low-risk 64.8% versus high-risk 64.4% in CUM, low-risk 76.5% versus high-risk 66% in UKH). In *Fig 2C,* we plotted the proportion of admissions with a given number of movements for all admissions with at least one movement on a log-linear scale.

When plotting admission LOS against mean number of movements per admission (*Fig 2D*), we observed a positive correlation in all participating hospitals, indicating that a patient with longer LOS is more likely to move more frequently between the departments during the hospital stay. To identify differences between the risk groups for the data shown in *Fig 2D*, we plotted number of movements against LOS for every hospital admission in each risk group for every hospital. There seems to be no clear difference between the risk groups for every hospital.

### Characteristics of intra-hospital movement networks

**Fig 3:**
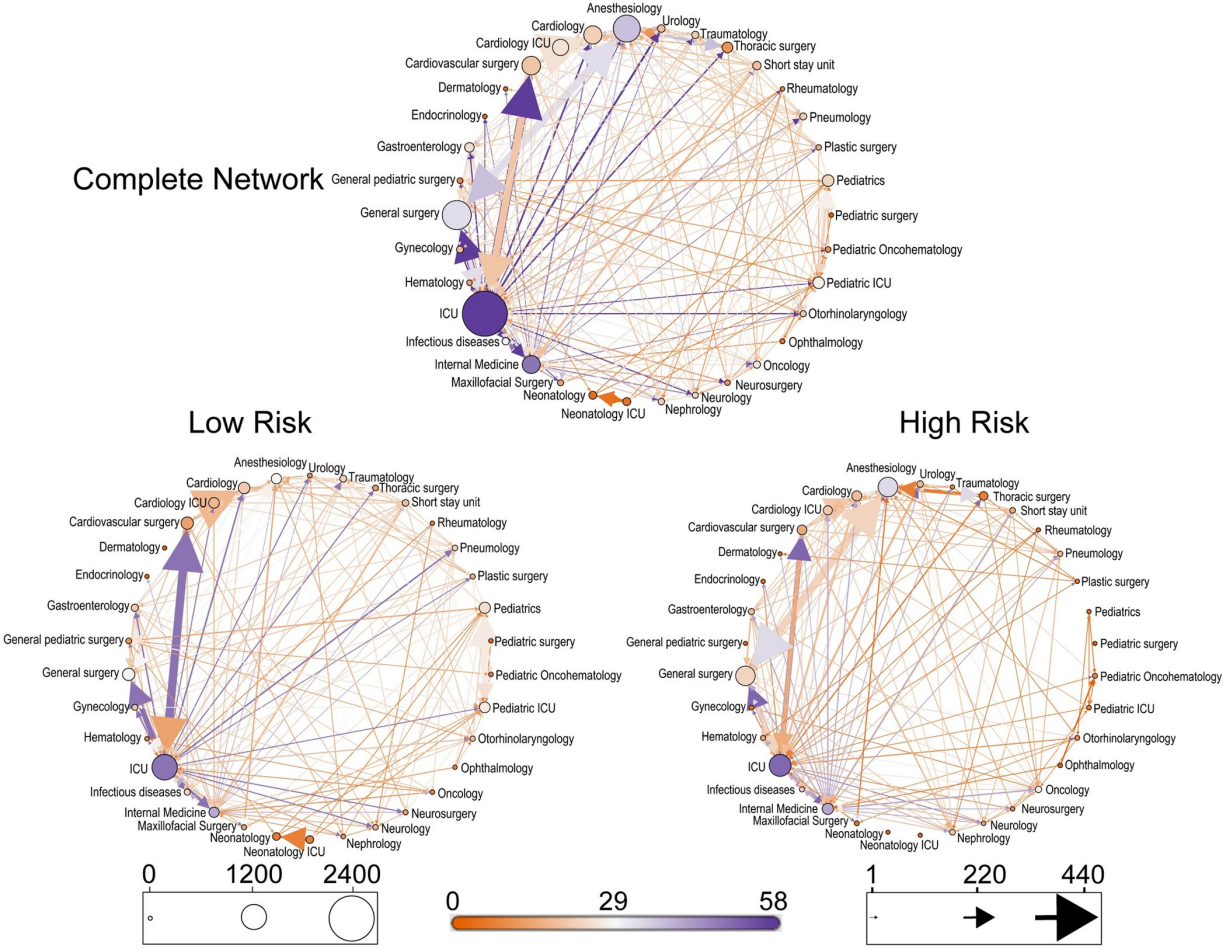
Intra-hospital hospital networks of HUVM showing patient directed movements from one department to another. Nodes represent departments and arrows represent patient movements between these departments. Color of the nodes was based on nodes degree whereas size of the nodes was based on nodes weighted degree. Arrows thickness is based on the directed number of transfers (weights) between the departments and color of an arrow is assigned similar to the node color from where the arrow is originating. For visualization of the clustering, a clustering layout of the complete HUVM network is also shown in the supplementary S1 Fig.

The intra-hospital movements were visualized as a weighted directed network, where nodes represent departments in a hospital and links are defined by patient flows. Directed weights of the links are based on the number of patient movements in each direction. Degree of a department is defined as the number of its connections to other departments. Weighted degree indicates the number of patients moving in and out from one department to other departments. *Fig 3* shows the visualization of the HUVM intra-hospital movement networks for both risk groups as well as for the complete data without stratification. Nodes were ordered alphabetically based on node names in a counter clockwise direction from the top node. The color of the node is based on node degree (sum of in-degree and out-degree), whereas the size of the node is based on node weighted degree (sum of weighted in-degree and weighted out-degree). The width of the link is based on weights (number of patient movements), where the thickness of a link is based on the number of patients moving in that direction. A clustering layout of the HUVM network is also shown in *S1 Fig*. Moreover, the number of patient movements between the departments are visualized as a heatmap (*S1 Fig*). The hospital movement networks for other considered hospitals are shown in *S2–S9 Figs*. Networks displayed in *Figs 3*and *S2–S9*clearly show that these networks had several central hubs with much incoming and outgoing patient movements, and many peripheral nodes that were only loosely connected to the network. The most common departments to act as hubs for both risk groups include ICU, emergency department, internal medicine, anesthesiology, cardiology and cardiothoracic surgery, neurology, cardiovascular surgery, and nephrology. However, pediatric departments tended to appear in the top few nodes for all networks in the low-risk groups except for BH, where no pediatric patients were admitted due to the absence of a pediatrics specialty.

To better compare the networks, we computed network statistics as shown in *Table 4*(see supplementary *S1 Text* for methods of calculating the reported network statistics). Number of edges and average degree were higher in the high-risk groups except HUVM and CUM where an opposite trend was observed. When compared to low-risk groups, slightly higher maximum degrees (degree range) were observed for high-risk groups for all hospitals. There were no clear relationships observed in the weighted degree, network diameter, and average path length between the risk groups and between the hospitals as these characteristics largely depend on the number of movements in each risk group of every hospital. *Table 4*also shows that average clustering coefficients in the high-risk groups were slightly higher except for HUVM. Among hospitals, the largest average clustering coefficients in both risk groups were observed in the BH. Similarly, graph density, showing the completeness and connectedness of a network, was higher in the high-risk groups for all hospitals except HUVM and CUM, where it was slightly lower than the density of the low-risk group network. BH showed relatively high graph density in both risk groups when compared to other hospitals (*Table 4*).

**Table 4:** Intra-hospital hospital networks statistics for each risk group. Complete data correspond to unstratified data including low-risk and high-risk groups.

Hospitals		UMCU			HUVM			CUM			BH			UKH		
		Complete	Low-risk	High-risk	Complete	Low-risk	High-risk	Complete	Low-risk	High-risk	Complete	Low-risk	High-risk	Complete	Low-risk	High-risk
Nodes		59			34			56			24			56		
Edges		1026	703	729	335	252	218	1219	1004	904	435	370	375	1076	720	871
Graph Density		0.3	0.205	0.213	0.298	0.225	0.194	0.396	0.326	0.294	0.788	0.67	0.679	0.349	0.234	0.283
Degree	Mean	17.39	11.92	12.356	9.85	7.41	6.41	21.77	17.93	16.14	18.13	15.42	15.62	19.21	12.86	15.55
	Range (min—max)	1–86	0–76	0–79	2–58	0–48	0–50	8–74	7–62	0–66	16–46	11–42	10–46	1–82	1–65	0–78
Avg. Weighted Degree per year		235.35	150.6	83.14	158.54	85.63	81.12	1118.6	723.14	366.48	63.83	31.40	32.43	340.80	172.73	168.07
Network Diameter		3	5	4	4	4	4	3	3	4	2	2	2	4	5	5
Avg. Clustering Coefficient		0.641	0.564	0.588	0.595	0.507	0.494	0.566	0.52	0.554	0.841	0.767	0.789	0.592	0.479	0.53
Avg. Path Length		1.759	1.968	2.03	1.852	1.962	1.855	1.644	1.757	1.857	1.212	1.332	1.321	1.769	2.03	1.884

### Simulation results

**Fig 4:**
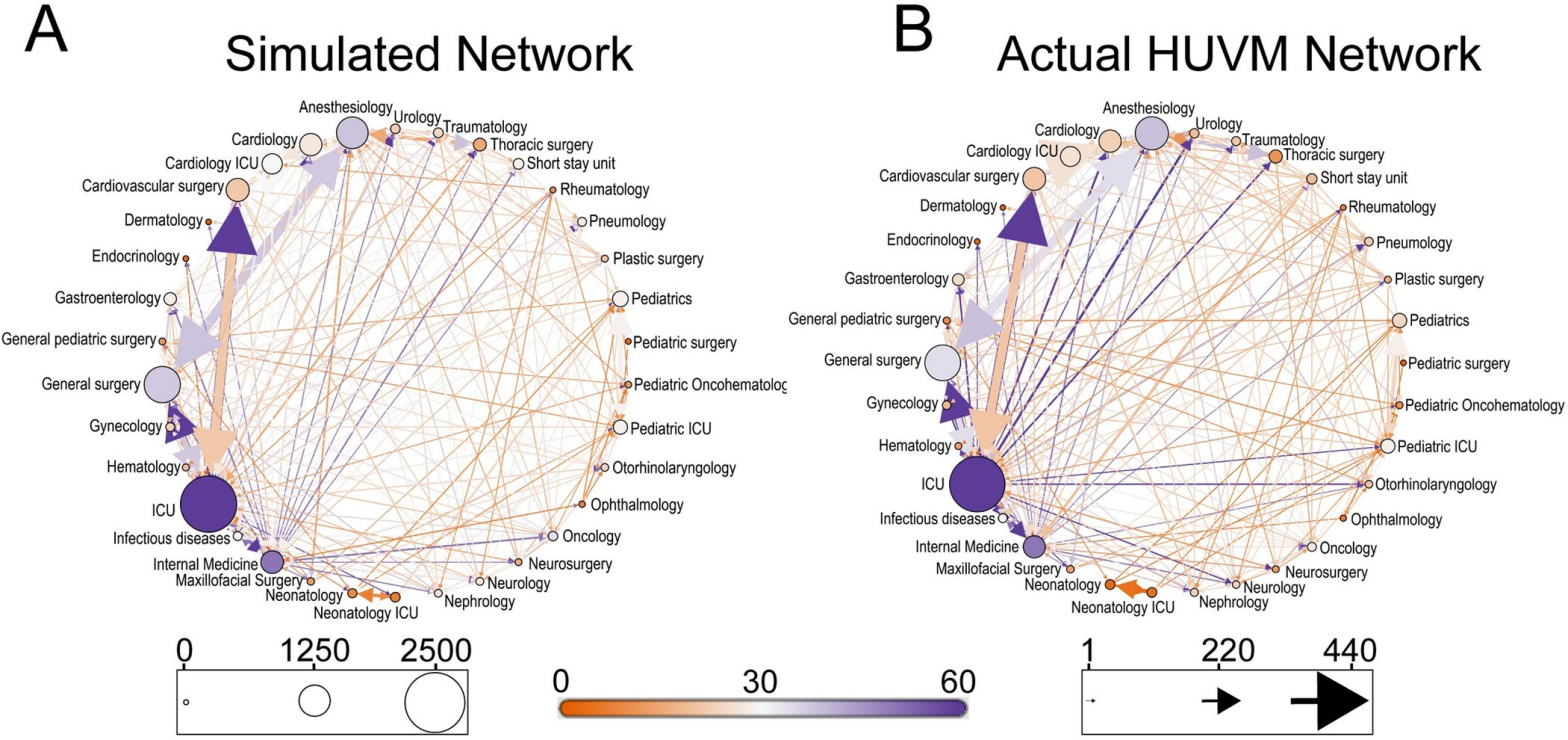
(A) Patient movements network generated from the ABM simulation, (B) Actual HUVM patient movement network without stratification of patients into low-risk and high-risk. Nodes represent departments and arrows represent patient movements between these departments. Color of the nodes is based on nodes degree and dark orange color refers to low values of degree whereas purple color refers to high degree. Size of the nodes is based on nodes weighted degree.

Based on patient movement patterns, we investigated the impact of movement network structure on pathogen spread. To do that, we used an agent-based model parameterized with data from the HUVM hospital. *Fig 4A* represents a network of patient movements averaged over 50 simulations. Visually, this network matches well with the un-stratified network of the HUVM (*Fig 4B*). *Fig 4A* shows the large variation in weighted degree of the nodes (departments), where the ICU acts as the biggest hub with highest weighted degree compared to other departments.

**Fig 5:**
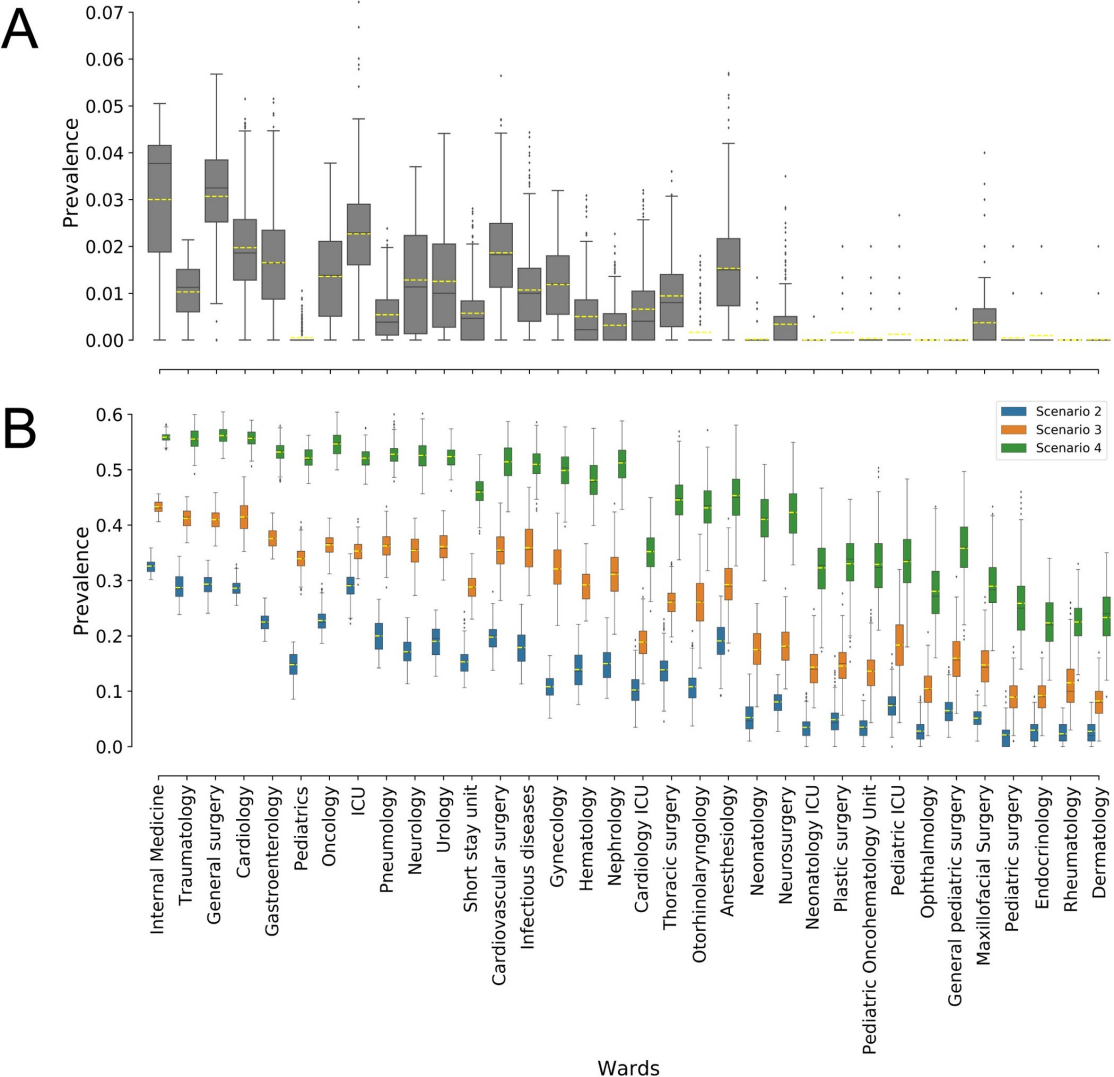
(A) Distribution of daily departments’ prevalence of MDR-E for a period of 395 days for scenario 1 where a single colonized patient was admitted to the ICU on day 30. (B) Distribution of steady state daily departments’ prevalence of MDR-E (from 200 days onwards) for scenario 2, 3 & 4 which include 1%, 5% and 15% daily arrivals of colonized patients respectively from day 30 onwards. Yellow lines represent the mean prevalence per department for every scenario. For all results shown in Fig 5, transmission parameter β = 0.25 was used for all departments. Departments are ordered by size with internal medicine department being the largest department. Results shown in Fig 5 are based on 50 simulations for every scenario.

For each scenario, distributions of department specific daily MDR-E prevalence (calculated as sum of colonized and infected patients divided by the total number of patients in a department) are plotted as box plots in *Fig 5*. Although we ran simulations with different values of *β* (*S1A Text*), we only showed results from *β =* 0.25 in *Fig 5*.

It is clear from *Fig 5A* that when MDR-E pathogen transmissions occurred in a highly-connected ICU department (*scenario 1*), it affected all the major departments of the hospital connected via patient movements. Although *scenario 1* was a hypothetical scenario with only a single admission of a colonized patient, it highlights the impact of patient movements on pathogen spread (*Fig 5A*). In reality, hospitals continuously receive colonized patients at admission. *Fig 5B* shows a steady state department specific daily prevalence of MDR-E pathogen for *scenarios 2*, *3*, *and 4*, which consider 1%, 5% and 15% of daily arrivals being colonized at admission, respectively. *Fig 5B* clearly illustrates that an increase in the percentage of colonized patients at admission has a direct impact on the departments’ MDR-E prevalence. Scenarios shown in *Fig 5B* show quite high prevalences in the larger and high centrality departments such as ICU, internal medicine, general surgery, cardiology and cardiovascular surgery. When the percentage of incoming colonized patients is low (1%, *scenario 2*), stochastic variations in departments’ mean prevalence are large for the large departments. However, with the increase in daily colonized arrivals (15% *scenario 4*), the majority of the large departments show similar levels of MDR-E mean prevalence.

**Fig 6:**
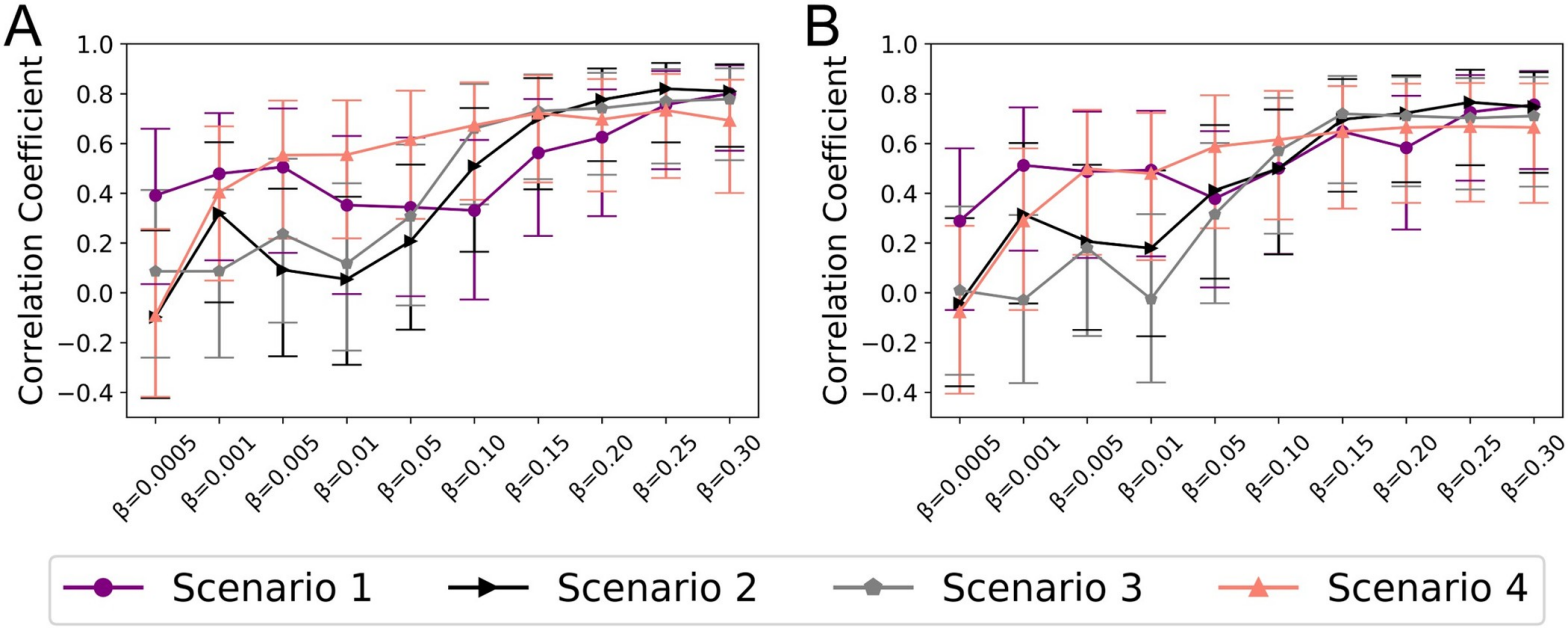
Spearman’s rank correlation coefficient results between averaged network statistics from 50 simulations and departments’ prevalence of MDR-E. (A) Correlation between departments’ degree and departments’ mean prevalence. (B) Correlation between departments’ weighted degree and departments’ mean prevalence. In all scenarios, different transmission parameter β values were tested. For Scenario 1, mean prevalence over a period of 395 days was used. For Scenario 2–4, steady state mean prevalence from 200 days onwards was used. Joining datapoints with lines was only done to improve readability but it does not show a functional relationship as x-axis is a categorical axis. For both A and B, errorbars represent 95% confidence intervals.

In order to quantify the impact of network characteristics on department specific MDR-E mean prevalence, we calculated Spearman’s rank correlation coefficients between departments’ MDR-E mean prevalence and network characteristics such as nodes degree (*Fig 6A*) and nodes weighted degree (*Fig 6B*) for different transmission rates (*β)* and for every scenario presented in *Fig 5*. *Fig 6A* and *6B* show that when a colonized patient is admitted to a high-centrality ICU department (*scenario 1*), departments’ prevalences showed a positive correlation with degree and weighted degree centralities respectively for all values of *β*. The correlations were much stronger for high *β* values (*β* > 0.10). For scenarios with continuous inflow of colonized patients (*scenarios 2–4*), strong associations between departments’ prevalences and both network degree and weighted degree were observed for *β* > 0.05 (*Fig 6A and 6B*). At extremely low *β* value (*β* = 0.0005), no association between departments’ prevalences and network centralities was observed for *scenarios 2–4*. At low *β* values (0.001 ≤ *β* ≤ 0.05), *scenarios 2 and 3* showed weak correlations, however, *scenario 4* did show an increasing trend between *β* values (0.001 ≤ *β* ≤ 0.05) and correlation coefficients.

Considering our modeling results and assuming no interventions in place, Table 5 indicates the top three departments with highest mean prevalence expected in the event of MDR-E spread for the considered hospitals, based on the above-mentioned network characteristics. Controlling patient movements out of these departments or applying other infection control interventions targeted to those high centrality departments may prevent MDR-E from spreading to remaining departments of the hospital.

**Table 5:** Top three departments of each participating hospital with simulated high mean prevalence based on network characteristics with respect to a hospital-wide MDR-E spread in the absence of interventions.

Hospital	Degree	Weighted Degree
**UMCU**	ICU, Neurology, Internal Medicine	ICU, Cardiothoracic surgery, Pediatrics ICU
**HUVM**	ICU, Internal Medicine, Anesthesiology	ICU, General surgery, Anesthesiology
**CUM**	CVK Anesthesiology, CVK Nephrology/Internal Intensive Care, CCM Anesthesiology	CBF Emergency Department, CVK Internal Emergency Department, CVK Anesthesiology
**BH**	Internal medicine D, Internal medicine A, Internal medicine C	Internal medicine D, Internal medicine E, Internal medicine B
**UKH**	ICU, Anesthesiology 1, Anesthesiology 2	Interdisciplinary emergency, ICU, Cardiac surgery 1

### Impact of intervention

**Fig 7:**
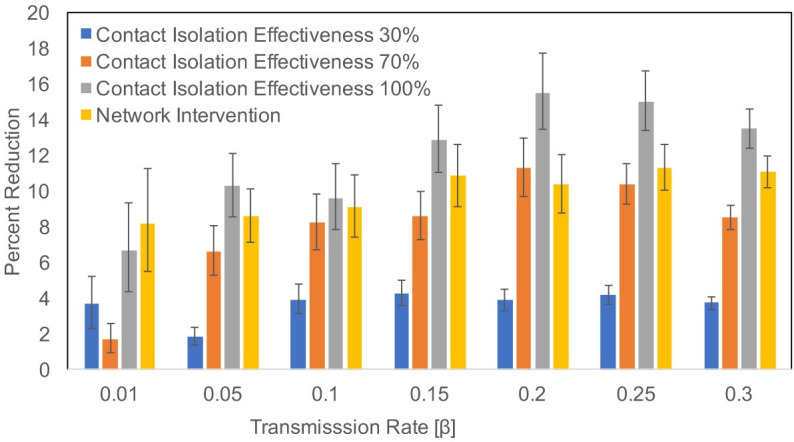
Impact of interventions on transmissions percent reductions for different β values. Error bars represent 95% confidence intervals.

We used the model to assess the impact of two interventions: (i) contact isolation of infected patients where we assumed that contact isolated patients were placed in separate rooms and their contacts with HCWs were reduced. We also assumed that HCWs were required to wear gloves and gowns and to follow strict hand hygiene protocols when entering contact isolated patients’ rooms. Depending on the effectivenss of these measures, contact isolated patients contributed less towards transmissions in the hospital. For this intervention, we tested different contact isolation effectiveness scenarios (30%, 70% and 100%), (ii) a network intervention where patients moving in and out from the highest (weighted) degree department (ICU) were screened (see Table 5). If a patient was detected as positive, the patient was put on contact isolation (assuming 90% effectiveness of contact isolation) for the remaining hospital stay. We calculated the percent reduction in the number of transmissions from the baseline scenario, where no intervention was applied. Fig 7 shows the impact of both interventions as percent reduction in the number of transmissions in the hospital over a period of 395 days for different transmission rates (β). We observed a clear impact of the contact isolation effectiveness where 100% effectiveness resulted in larger reductions in number of transmissions as compared to 30% and 70% contact isolation effectiveness. Depending on the transmission rate (β), the network intervention applied to just one department with the highest degree and weighted degree resulted in 8–11% reduction in the number of transmissions. Results for extremely low β values (β < 0.01) are not plotted in Fig 7 because the number of transmissions were very low and percent reduction results did not make any sense. Fig 7 also shows that a contact isolation of infected patients which is 100% effective is best, but if contact isolation is not perfect, a network based approach may be better. Instead of targeting just one highest degree and weighted degree department, extending the network intervention to include several departments may result in more reductions in the number of transmissions in the hospital.

## Discussion

Our study provides a detailed description of patterns of patients’ movements between departments in a hospital based on data from several hospitals, and an analysis of how these movements may impact the transmission of bacterial pathogens within hospitals.

Our analysis includes a stratification by patients’ risk levels and its implications for patients’ movements and risk of becoming colonized. Risk of acquiring colonization may differ between patients and may be determined by several factors such as functional status, immune responses, chronic or severe diseases. Taking such factors into account, we stratified patients into low-risk and high-risk groups based on certain ICD-10 codes. Results indicate that high-risk patients were on average slightly older and stay longer in the hospital than low-risk patients. High-risk patients moved between departments slightly more often per admission than low-risk patients; however, this higher number of movements may be due to their longer hospital stay. We further visualized patient intra-hospital movement patterns from participating hospitals as networks, and used an ABM to further assess the impact of intra-hospital movements on pathogen spread. Our modeling results clearly show that a MDR-E spread in one of the high centrality departments can spread out to all the departments in a hospital. Positive correlations between departments’ centralities and departments’ mean prevalence show that departments with high centralities will eventually have high prevalence at higher transmission rates. Therefore, consideration of departments’ centralities and patient movements in a given hospital could improve the efficiency of interventions.

Patient movements inside the hospital have been included in few modeling studies for MRSA and MDR-E pathogens [18–20] but those studies have considered only a few specialty departments and the departments’ structure (e.g. different department sizes) is often neglected. Rocha et al. [37] and Pei et al. [38] have recently studied MRSA spread in a hospital network where they developed a large scale data-driven contact network model including the dynamics of patient referrals within and between wards and hospitals. Since their model captures the interaction patterns that were formed from time varying real-world contact data, they did not explicitly consider the department types and structure of hospitals. Here we identified, based on data from specific hospitals and data on network centrality, which departments may play a crucial role in the spread of MDR-E in the entire hospital. In the absence of interventions, simulation results showed a strong positive correlation between departments’ mean prevalence and network characteristics such as degree and weighted degree.

### Study limitations

Disparities in the department structure exist between hospitals, e.g., a hospital could have two departments that might be a single department in another hospital. Every hospital structure is different and a generalization of the hospital structure is therefore not possible. For the hospitals included in our study, the German hospitals (CUM and UKH) did include data from emergency departments, but other hospitals did not include emergency departments in their data set. Such differences in the hospital structures and data sets made it much harder to compare these hospitals with each other. Moreover, the CUM hospital is a very large hospital in Berlin which has three separate campuses across Berlin. Although it would also be possible to consider these CUM campuses as separate hospitals, we did observe substantial patient movements between these campuses. Furthermore, the BH did not have a pediatrics department, and ICU data were stored in a separate database, which was not provided for the analysis.

In the current work, movement networks were created from routine patient data. It could be that some patient movements in the datasets were not physical movements, but represented administrative events, when a patient received treatment from another department, while staying in the same place. We are aware of this issue, however we did not have sufficient information to filter out such movement records from the datasets. A more accurate way of tracing patient movements between the departments of a hospital would be to use wireless wearable sensors as used in the close proximity interaction studies [39–41], however, gathering data with such methods is often limited to shorter time periods given the high costs and privacy protection issues.

In the presented model, upon a successful transmission, a patient is assumed to become immediately colonized, and starts transmitting the pathogen to others, which may not be the case in reality. In future work, a latent period may be included to allow some time delay for a patient to acquire enough bacterial load before transmitting to others. We do not expect strong impact of a latent period on the correlation results, but a latent period might affect prevalence levels. Moreover, it can be considered to include a direct pathway from *susceptible* to an *infected* disease state.

### Implications for clinical practice

In the current study, patient movements between the departments of a single hospital were extracted from anonymized hospital admission data. Such data is stored in almost every hospital information system. This work highlights the potential of using such data to evaluate patient movement patterns and their implications for pathogens spread inside the hospital.

When an infectious disease is severe, large changes in the patient movement patterns may become necessary, and will be implemented as witnessed during the COVID-19 pandemic. However, changing patient movements in a hospital to prevent spread of pathogens such as MDR-E may not be a viable option. In view of our results about correlation between network characteristics and prevalence, one could consider establishing a ranking system based on departments’ network characteristics, such as degree and weighted degree. In such a ranking system, departments are sorted by their degree and weighted degree. When the colonization status of patients coming from different departments is unknown, a risk assessment may be based upon the rank of the department the patient is coming from. If the respective department has a high rank, the patient may be placed in isolation or increased hygienic measures could be taken as a precautionary infection control measure. The advised intervention should only be applied when there are available resources, in terms of free beds available in the department. Moreover, priority should be given to patients in need of urgent medical care and after that, if resources are still available, patients coming from different departments can be handled based on the movement ranking system. This may prevent spread of pathogens among departments that are connected via patient movements.

To conclude, our study emphasizes the importance of intra-hospital patient movements and their impact on pathogen spread. Applying interventions by targeting hubs, i.e. departments of higher degree and weighted degree centrality may help to control the spread of MDR-E. Moreover, when the colonization status of patients coming from different departments is unknown, a department ranking system based on centrality measures could be used to improve the efficiency of the interventions.

## Supporting information

S1 Text Agent Based Intra-hospital model. (DOCX)

S1 Fig (A) Inter-department complete HUVM hospital network showing clustering of the departments. Clustering is computed based on the modularity algorithm in the Gephi software which detects nodes that are more densely connected together than to the rest of the network. Node colors show the cluster to which a node belongs. The color of the arrow is based on the color of the node from where the arrow is originating. The thickness of the arrow is based on the number of patient’s transfers (weight). The size of the node is based on the weighted degree. (B) Heat map showing the number of transfers from one department to another department for the complete HUVM network. A patient is transferred from the source to the target department.
(PDF)

S2 Fig Inter-department hospital networks of the UMCU hospital showing patient directed movements from one department to another. (A) Complete UMCU network without stratification, (B) Low-risk UMCU network, (C) High-risk UMCU network. Nodes represent departments and arrows represent patient movements between these departments. The color of the nodes was based on nodes degree whereas size of the nodes was based on the nodes’ weighted degree.
(PDF)

S3 Fig (A) Inter-department complete UMCU hospital network showing clustering of the departments. Clustering is computed based on the modularity algorithm in the Gephi software which detects nodes that are more densely connected together than to the rest of the network. Node colors show the cluster to which a node belongs. The color of the arrow is based on the color of the node from where the arrow is originating. The thickness of the arrow is based on the number of patient’s transfers (weight). The size of the node is based on the weighted degree. (B) Heat map showing the number of transfers from one department to another department for the complete UMCU network. A patient is transferred from the source to the target department.
(PDF)

S4 Fig Inter-department hospital networks of the CUM hospital showing patient directed movements from one department to another. (A) Complete CUM network without stratification, (B) Low-risk CUM network, (C) High-risk CUM network. Nodes represent departments and arrows represent patient movements between these departments. The color of the nodes was based on nodes degree whereas size of the nodes was based on nodes weighted degree. CBF, CCM and CVK are different campuses of the CUM hospital.
(PDF)

S5 Fig (A) Inter-department complete CUM hospital network showing clustering of the departments. Clustering is computed based on the modularity algorithm in the Gephi software which detects nodes that are more densely connected together than to the rest of the network. Node colors show the cluster to which a node belongs. The color of the arrow is based on the color of the node from where the arrow is originating. The thickness of the arrow is based on the number of patient’s transfers (weight). The size of the node is based on the weighted degree. (B) Heat map showing the number of transfers from one department to another department for the complete CUM network. A patient is transferred from the source to the target department.
(PDF)

S6 Fig Inter-department hospital networks of the BH hospital showing patient directed movements from one department to another. (A) Complete BH network without stratification, (B) Low-risk BH network, (C) High-risk BH network. Nodes represent departments and arrows represent patient movements between these departments. The color of the nodes was based on nodes degree whereas size of the nodes was based on the nodes’ weighted degree.
(PDF)

S7 Fig (A) Inter-department complete BH hospital network showing clustering of the departments. Clustering is computed based on the modularity algorithm in the Gephi software which detects nodes that are more densely connected together than to the rest of the network. Node colors show the cluster to which a node belongs. The color of the arrow is based on the color of the node from where the arrow is originating. The thickness of the arrow is based on the number of patient’s transfers (weight). The size of the node is based on the weighted degree. (B) Heat map showing the number of transfers from one department to another department for the complete BH network. A patient is transferred from the source to the target department.
(PDF)

S8 Fig Inter-department hospital networks of the UKH hospital showing patient directed movements from one department to another. (A) Complete UKH network without stratification, (B) Low-risk UKH network, (C) High-risk UKH network. Nodes represent departments and arrows represent patient movements between these departments. The color of the nodes was based on nodes degree whereas size of the nodes was based on the nodes weighted degree.
(PDF)

S9 Fig (A) Inter-department complete UKH hospital network showing clustering of the departments. Clustering is computed based on the modularity algorithm in the Gephi software which detects nodes that are more densely connected together than to the rest of the network. Node colors show the cluster to which a node belongs. The color of the arrow is based on the color of the node from where the arrow is originating. The thickness of the arrow is based on the number of patient’s transfers (weight). The size of the node is based on the weighted degree. (B) Heat map showing the number of transfers from one department to another department for the complete UKH network. A patient is transferred from the source to the target department.
(PDF)

S10 Fig Mean number of patients present per day in every department of the HUVM hospital. This data was used to define department size in terms of beds per department.
(PDF)

S11 Fig Daily inter-department discharge rates from every department in the HUVM hospital. Departments are ordered by department size as shown in S10 Fig.
(PDF)

S12 Fig Patient disease state flow chart. S refers to Susceptible, C refers to Colonized, and I refers to symptomatic infected patients. FOI is the force of infection.
(PDF)

S13 Fig Impact of additional LOS for infected patients on steady state prevalence using Scenario 3 (5% continuous arrival of colonized patients). Transmission parameter *β* = 0.25 was used in each department.
(PDF)

S14 Fig Spearman’s rank correlation coefficients between departments prevalence and network characteristics (degree and weighted degree). Different values for increase in the LOS for infected patients were used to identify the impact of this parameter on the correlation between steady state departments prevalence and network characteristics. Scenario 3 (5% continuous arrival of colonized patients) with transmission parameter *β* = 0.25 for every department was used.
(PDF)

S15 Fig Impact of additional LOS for infected patients on the overall hospital LOS distributions in both risk groups (Low-risk and High-risk) and its comparison with the actual HUVM data for both risk groups. Grey lines in the boxes show median of the data.
(PDF)

S1 Numerical Data Numerical data of Fig 1C. (XLSX)

S2 Numerical Data Numerical data of Fig 2D. (XLSX)
