# Supplementary material for: Relevance of intra-hospital patient movements for the spread of healthcare-associated infections within hospitals - a mathematical modeling study
Source: PLoS Comput Biol. 2021 Feb 3;17(2):e1008600. doi: 10.1371/journal.pcbi.1008600 (PMC7857595; doi:10.1371/journal.pcbi.1008600)
Supplement: S1 Text — (DOCX) [file pcbi.1008600.s001.docx]

**S1 Text: Agent Based Intra-hospital model**

An agent based model (ABM) has been developed to simulate the spread of multidrug resistant enterobacteriacae (MDR-E) pathogens inside the hospital. In the current model, we explicitly simulated only patients. Transmission between patients occurred via the cross-transmission route, which involved effective contact between patients and health care workers (HCWs).

To initiate simulations, the user had to define the percentage of free beds (from 0% – 100%) at the start of the simulation. Based on this given percentage, beds from every department were kept free at the start of the simulation ($t_{0}$)*.* Patients were then admitted to all remaining available beds in the hospital at $t_{0}$. In the simulations presented in this paper, we chose this parameter to be 20% at $t_{0}$. Based on the daily arrival rate of new patients into the hospital, the patient population in the hospital become stable within the first few days.

In addition, we needed to define the number of departments and beds per department in the model. There were 34 departments in the HUVM dataset. The number of beds in every department were estimated from the HUVM dataset by counting mean number of patients present every day in a department. *S10 Figure* shows the mean number of patients present every day in each department.

Every patient has a risk score (high-risk or low-risk) which was assigned to every patient at the time of admission. Since we observed in the HUVM dataset that on average, 36.69% patients belong to the high-risk group, we used a probability of 0.3669 for the patients to arrive as high-risk patients (at $t_{0}$). If a patient was not classified as high-risk at admission, it was automatically considered as a low-risk patient. Both low-risk and high-risk patients were uniformly distributed to all departments in the hospital. The main difference between the low-risk and high-risk patients was in the length of stay (LOS) of these patients during their hospital admission. Depending on the risk group of a patient, a LOS in discrete time units was assigned to every individual agent from an exponential probability density function (estimated from the HUVM dataset separately for both risk groups).

Patients were also characterized by their disease state: *susceptible*, *colonized*, or *infected*. A patient in the *susceptible* state can immediately become colonized after being exposed to MDR-E pathogens. A patient in a *colonized* state remains asymptomatically colonized with MDR-E and can transmit the pathogen to others upon contact. A patient in an *infected* state is symptomatically colonized showing disease symptoms. Furthermore, infected patients can still spread the pathogen to others. The majority of the patients admitted to the hospital come in a *susceptible* state and depending on the scenario, few come in a *colonized* state. Parameters used in the current model are shown in *Table A*.

***Run time of the model***

*New patients’ arrival:*

New patients come to the hospital on a daily arrival rate according to a Poisson distribution. If new patients needed to be admitted into the hospital, our model assessed the availability of departments with vacant beds. If vacant beds were available, patients were uniformly distributed in the hospital departments and all the properties such as LOS, disease state, risk score, etc. were assigned. If all beds were occupied, patients were added to a queue and waited to be assigned to an available bed. From the daily new patient arrivals, we used a probability of 0.3669 for a patient to arrive as a high-risk patient.

*Patient discharge:*

Since LOS is assigned in discrete time units, every patient LOS was continuously counted down during the run time. Once the LOS of a patient reached zero, the patient was discharged from the hospital by being removed from the agent list.

*Patient movement:*

In the data, for patients who made two or more movements in the HUVM hospital, we observed that in 81% of such movements, patients were moved back to the previous ward. Based on this observation, we also implemented in the ABM that for patients who made two or more movements, there is an 81% chance that they will return to the previous ward. For the remaining 19% of the movements, patients follow the below explained department selection algorithm using a preference matrix. For every department of the HUVM hospital, daily patient movement rates to other departments were estimated (*S11 Figure*). These movement rates were then divided by the size of the departments to obtain department-specific daily movement probabilities. Given those daily movement probabilities, numbers of patients to be moved to other departments were calculated for every department every simulated day, and added to a department specific counter. This counter kept track of the number of patients to be moved from every department. If there was a patient that needs to be moved from a department, the next step was to find a future department where patient could go. This was implemented using a preference matrix composed of preference probabilities [1]. For this preference matrix, a pivot table was first computed based on every department’s weighted in-degree and weighted out-degree from the HUVM hospital. This pivot table was then normalized row-wise to obtain preference probabilities. Given those preference probabilities [1], a patient’s next department was selected and the patient was moved to the new department. It is worthwhile to note, that there are fixed numbers of beds per department, which can therefore all be occupied at a certain moment. If no vacant bed was available in the new department, the above procedure of selecting a new department from the preference matrix is repeated every time step until a patient’s move to the new department is successfully completed.

**Table A**: Model parameters

| ***Parameter*** | ***Value*** | ***Details*** |
| --- | --- | --- |
| *1 Simulation step (t) [in minutes (real time)]* | *5* | *Assumed* |
| *Maximum simulation time (t_max_) [Days]* | *427 (incl. 30 days burn out period)* | *Based on HUVM data which has a data period of 395 days* |
| *Beds vacant at model initialization* | *20%* | *Assumed. Based on daily arrival of patients, hospital occupancy reaches an equilibrium in few days after start of the simulation.* |
| *Number of departments* | *34* | *Estimated from HUVM data* |
| *Beds per department* | *[135,42,38,36,35,25,23,22,22,18,*  *17,14,13,12,12,9,9,7,7,6,6,5,5,4,*  *3,3,3,3,3,3,3,2,2,2,2,2]* | *Estimated from HUVM data (S10 Figure)* |
| $\lambda_{lr}$  *(LOS parameter for low-risk patients)* | *0.221* | *Estimated from HUVM data; used an exponential probability density function to assign LOS to low-risk patients.*  $P_{lr}\left( X \right)=\lambda_{lr}e^{-\lambda_{lr}x} 1\leq x \leq100$ |
| $\lambda_{hr}$  *(LOS parameter for high-risk patients)* | *0.150* | *Estimated from HUVM data; used an exponential probability density function to assign LOS to high-risk patients.*  $P_{hr}\left( X \right)=\lambda_{hr}e^{-\lambda_{hr}x} 1\leq x \leq100$ |
| *Average patient arrival rate [patients/day]* | *70* | *Estimated from HUVM data* |
| *High risk patient’s daily arrival probability* | *0.3669* | *Estimated from HUVM data* |
| *colonized patient’s daily arrival probability* | *1%, 5%, 15%* | *Depending on the scenario. Assumed and tested various values* |
| *Transmission rate* $\beta$ *[per day]* | *0.0005, 0.001, 0.005, 0.01, 0.05, 0.10, 0.15, 0.20, 0.25, 0.30* | *Assumed, tested various values. Transmission rates do not differ between departments.* |
| *Infected patients additional increase in LOS [Days]* | *0, 1, 2, 3, 4, 5, 6, 7* | *Assumed and tested different values (S13 and S14 Figures). We used 3 days increase in the LOS for simulations presented in this paper.* |
| *Duration of infection [Days]* | *0,1, 2, 3, 4, 5, 6, 7* | *Assumed and tested different values (S13 and S14 Figures). We used 3 days as a default duration of infection for simulations presented in this paper.* |
| *Probability colonized to infected [per day]* | *0.012* | *This value results in approx. 5% infected patients in the model as mentioned by Martin et al. [2]* |

*State Change:*

Every patient also has a disease state. A patient state was updated every 24 hours. A patient state change diagram is shown in *S12 Figure*. Transmission between patients occurred via the cross-transmission route which involves contact between patient and HCWs. Since we did not model HCWs explicitly, transmission was implemented as a force of infection which gives the probability per unit time *t* for a susceptible patient to acquire the pathogen and to become colonized.

The force of infection (*FOI*) is calculated by the following equation:

$$FOI=\beta\frac{C\left( t \right)+I\left( t \right)}{N\left( t \right)}$$

$\beta$ is the transmission parameter. $C\left( t \right)$, $I\left( t \right)$ and $N\left( t \right)$ represent the number of colonized patients, number of infected patients and the total number of patients present in a department respectively. Every 24 hours, we use the calculated *FOI* as a probability in the Bernoulli trail for every susceptible patient in a department and, if the Bernoulli trial is successful, a patient state is changed from *susceptible* to *colonized*. A fraction of colonized patients can become infected and in the current model, we assume that there is a 0.012/day probability that a colonized patient can become infected. We use this probability to do a Bernoulli trial and if the trial is successful, we change the patient state to *infected* otherwise the patient remains colonized. This results in a cumulative percentage of approx. 5% infected patients in the model as reported by Martin et al. [2]. Once a patient state is changed to *infected*, its LOS is increased by 3 days (in discrete time units). After the end of this extra time, the patient state is changed back to *colonized*. Depending on the scenario, there is a continuous influx of colonized patients in the model.

***Effect of LOS increase for infected patients:***

In *Table A* and *S12 Figure*, colonized patients move to infected state and their LOS is increased by 3 days. After this additional time is over (duration of infection), the patient state is turned to a *colonized* state. This parameter is pathogen specific and depends also on the severity of infection and is, therefore, difficult to estimate. We performed a sensitivity analysis for this parameter and assessed its impact on the steady state department prevalence using *Scenario 3* (5% continuous colonized patient arrivals), shown in *S13 Figure*. It is clear from *S13 Figure* that the increase in the LOS for infected patients has a substantial impact on department prevalence. The prevalence in a department increases with the increase in the additional LOS parameter. Despite this, we did not notice any impact of this parameter on the correlation between department prevalence and network characteristics such as degree and weighted degree (*S14 Figure*).

**Network Measures:**

**Degree:** For a directed network *G*, each node has two degrees. The in-degree (*K_i_^in^*) of a node *i* is the number of edges coming towards the node *i*. The out-degree (*K_i_^out^*) of a node *i* is the number of outgoing edges emanating from the node *i*. The total degree (*K_i_*) of a node *i* is the sum of *K_i_^in^* + *K_i_^out^*. The average degree of a directed network *G* can then be calculated as:

$$\frac{1}{N}\sum_{i}^{N} k_{i}^{in}= \frac{1}{N}\sum_{i}^{N} k_{i}^{out}=\frac{E}{N}$$

where *E* is the number of edges and *N* is the number of nodes.

**Weighted Degree**: In case of weighted degree of a directed weighted network, the node degree *k_i_* can be extended directly to the weighted degree (*S_i_*), which is the sum of weights of all the links attached to node *i*. Similar to degree, weighted degree has two weighted degrees: weighted in-degree (*S_i_^in^*) and weighted out-degree (*S_i_^out^*). *S_i_^in^* of a node *i* is the sum of the weights of edges coming towards node *i.* Similarly, *S_i_^out^* of the node *i* is the sum of the weights of edges emanating from the node *i*. The total weighted degree (*S_i_*) of a node *i* is the sum of *S_i_^in^* +*S_i_^out^*. The average weighted degree of a weighted network *G* is then:

$$\frac{1}{N}\sum_{i}^{N} S_{i}^{in}= \frac{1}{N}\sum_{i}^{N} S_{i}^{out}=\frac{1}{2N}\sum_{i}^{N} S_{i}$$

**Graph Density:** Graph density= number of edges / number of possible edges. For a directed network G, the number of possible edges can be calculated as: *N * (N-1*) where *N* is number of nodes. A fully connected network has a graph density of 1.0.

**Network Diameter**: The network diameter *D* of a network having *N* nodes is defined as the maximum of the shortest paths between any two nodes in the network.

**Average Path Length:** Average path length *L_G_* is defined as the average number of steps along the shortest paths for all possible directed pairs of network nodes and is calculated as:

$$L_{G}=\frac{1}{c} \sum_{i \neq j} d(n_{i},n_{j})$$

where *d(n_i_,n_j_)* denotes the shortest distance between nodes *n_i_* and *n_j_*, *c* denotes the number of possible directed pairs. It is to be noted here that weights of the directed edges are not considered for the calculation of *L_G_*.

**Average Clustering Coefficient:** Clustering coefficient of a node in a network computes how close its neighbours are to being a clique (complete graph). In a directed network, clustering coefficient of a node *i* is calculated by the formula:

$$C_{i}=\frac{M_{i}}{k_{i}\left( k_{i}-1 \right)}$$

where *M_i_* is the number of directed links between the neighbours of the node *i*, and *k_i_* is the degree of the node *i*. The average clustering coefficient is the mean value of the individual clustering coefficient (*C_i_*) of every node *i* and is given by:

$$C_{G}= \frac{1}{N}\sum_{i}^{N} C_{i}$$

***References***

1. HUVM. Patient movement preference matrix, <https://www.dropbox.com/s/l9edlqwus2g0hmm/HUVM_preference_matrix.csv?dl=0>.

2. Martin RM, Bachman MA. Colonization, infection, and the accessory genome of Klebsiella pneumoniae. Frontiers in cellular and infection microbiology. 2018;8:4.
