# Supplementary material for: Relevance of intra-hospital patient movements for the spread of healthcare-associated infections within hospitals - a mathematical modeling study
Source: PLoS Comput Biol. 2021 Feb 3;17(2):e1008600. doi: 10.1371/journal.pcbi.1008600 (PMC7857595; doi:10.1371/journal.pcbi.1008600)
Supplement: S1 Fig — (A) Inter-department complete HUVM hospital network showing clustering of the departments. Clustering is computed based on the modularity algorithm in the Gephi software which detects nodes that are more densely connected together than to the rest of the network. Node colors show the cluster to which a node belongs. The color of the arrow is based on the color of the node from where the arrow is originating. The thickness of the arrow is based on the number of patient’s transfers (weight). The size of the node is based on the weighted degree. (B) Heat map showing the number of transfers from one department to another department for the complete HUVM network. A patient is transferred from the source to the target department. (PDF) [file pcbi.1008600.s002.pdf]

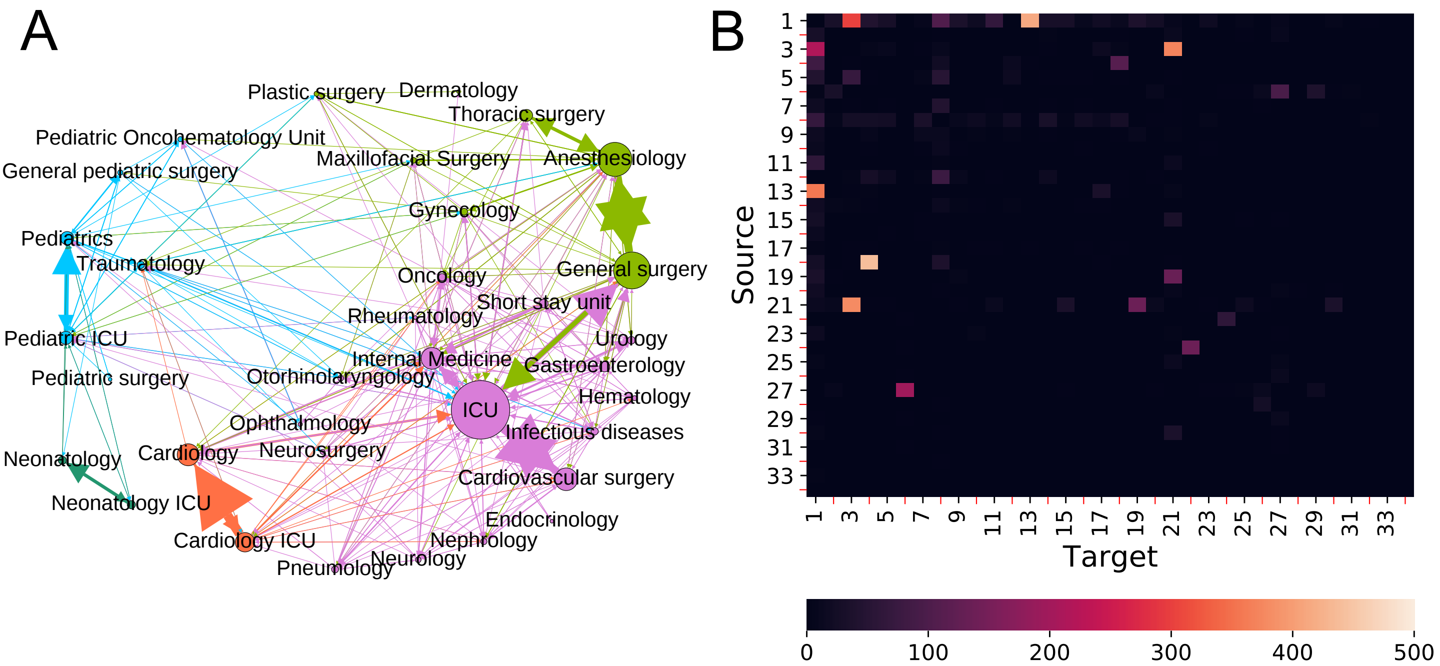

1 -ICU
2 -Traumatology
3 -General surgery
4 -Cardiology
5 -Gastroenterology
6 -Pediatrics
7 -Oncology
8 -Internal Medicine
9 -Pneumology
10 -Neurology
11 -Urology
12 -Short stay unit
13 -Cardiovascular surgery
14 -Infectious diseases
15 -Gynecology
16 -Hematology
17 -Nephrology
18 -Cardiology ICU
19 -Thoracic surgery
20 -Otorhinolaryngology
21 -Anesthesiology
22 -Neonatology
23 -Neurosurgery
24 -Neonatology ICU
25 -Plastic surgery
26 -Pediatric Oncohematology Unit
27 -Pediatric ICU
28 -Ophthalmology
29 -General pediatric surgery
30 -Maxillofacial Surgery
31 -Pediatric surgery
32 -Endocrinology
33 -Rheumatology
34 -Dermatology
