# Supplementary material for: Relevance of intra-hospital patient movements for the spread of healthcare-associated infections within hospitals - a mathematical modeling study
Source: PLoS Comput Biol. 2021 Feb 3;17(2):e1008600. doi: 10.1371/journal.pcbi.1008600 (PMC7857595; doi:10.1371/journal.pcbi.1008600)
Supplement: S2 Fig — (A) Complete UMCU network without stratification, (B) Low-risk UMCU network, (C) High-risk UMCU network. Nodes represent departments and arrows represent patient movements between these departments. The color of the nodes was based on nodes degree whereas size of the nodes was based on the nodes’ weighted degree. (PDF) [file pcbi.1008600.s003.pdf]

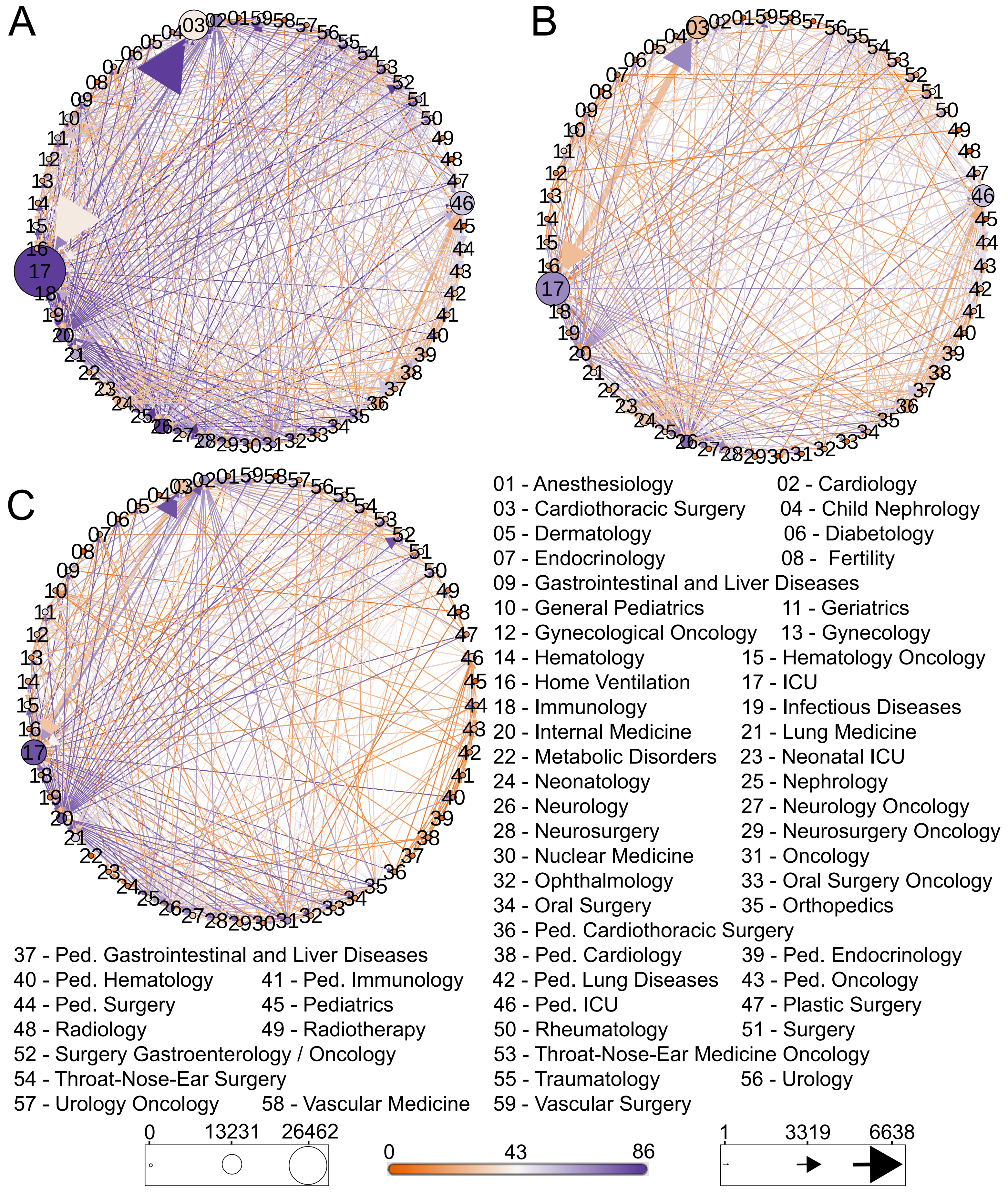

A
B
C
01 - Anesthesiology
02 - Cardiology
03 - Cardiothoracic Surgery
04 - Child Nephrology
05 - Dermatology
06 - Diabetology
07 - Endocrinology
08 - Fertility
09 - Gastrointestinal and Liver Diseases
10 - General Pediatrics
11 - Geriatrics
12 - Gynecological Oncology
13 - Gynecology
14 - Hematology
15 - Hematology Oncology
16 - Home Ventilation
17 - ICU
18 - Immunology
19 - Infectious Diseases
20 - Internal Medicine
21 - Lung Medicine
22 - Metabolic Disorders
23 - Neonatal ICU
24 - Neonatology
25 - Nephrology
26 - Neurology
27 - Neurology Oncology
28 - Neurosurgery
29 - Neurosurgery Oncology
30 - Nuclear Medicine
31 - Oncology
32 - Ophthalmology
33 - Oral Surgery Oncology
34 - Oral Surgery
35 - Orthopedics
36 - Ped. Cardiothoracic Surgery
37 - Ped. Gastrointestinal and Liver Diseases
38 - Ped. Cardiology
39 - Ped. Endocrinology
40 - Ped. Hematology
41 - Ped. Immunology
42 - Ped. Lung Diseases
43 - Ped. Oncology
44 - Ped. Surgery
45 - Pediatrics
46 - Ped. ICU
47 - Plastic Surgery
48 - Radiology
49 - Radiotherapy
50 - Rheumatology
51 - Surgery
52 - Surgery Gastroenterology / Oncology
53 - Throat-Nose-Ear Medicine Oncology
54 - Throat-Nose-Ear Surgery
55 - Traumatology
56 - Urology
57 - Urology Oncology
58 - Vascular Medicine
59 - Vascular Surgery
0
13231
26462
0
43
86
1
3319
6638
