# Supplementary material for: Relevance of intra-hospital patient movements for the spread of healthcare-associated infections within hospitals - a mathematical modeling study
Source: PLoS Comput Biol. 2021 Feb 3;17(2):e1008600. doi: 10.1371/journal.pcbi.1008600 (PMC7857595; doi:10.1371/journal.pcbi.1008600)
Supplement: S3 Fig — (A) Inter-department complete UMCU hospital network showing clustering of the departments. Clustering is computed based on the modularity algorithm in the Gephi software which detects nodes that are more densely connected together than to the rest of the network. Node colors show the cluster to which a node belongs. The color of the arrow is based on the color of the node from where the arrow is originating. The thickness of the arrow is based on the number of patient’s transfers (weight). The size of the node is based on the weighted degree. (B) Heat map showing the number of transfers from one department to another department for the complete UMCU network. A patient is transferred from the source to the target department. (PDF) [file pcbi.1008600.s004.pdf]

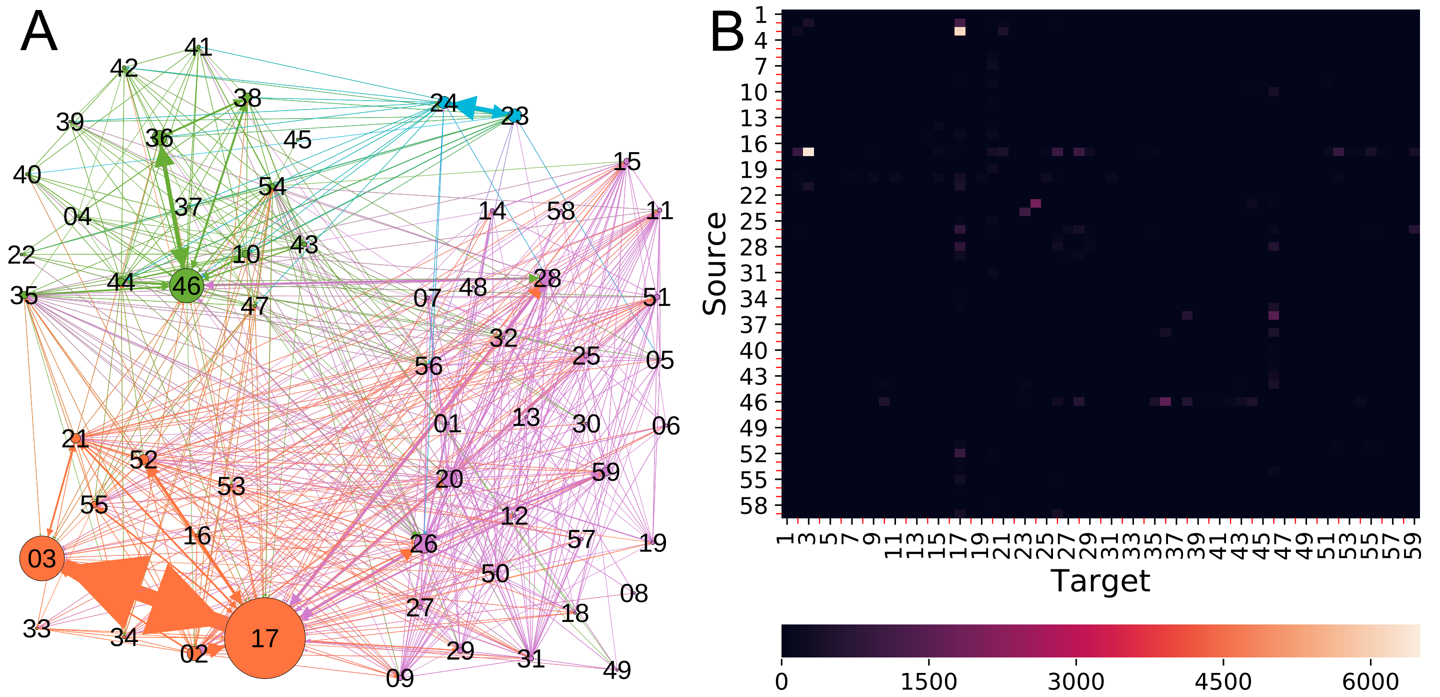

01 - Anesthesiology 02 - Cardiology 03 - Cardiothoracic Surgery 04 - Child Nephrology
05 - Dermatology 06 - Diabetology 07 - Endocrinology 08 - Fertility
09 - Gastrointestinal and Liver Diseases 10 - General Pediatrics 11 - Geriatrics
12 - Gynecological Oncology 13 - Gynecology 14 - Hematology 15 - Hematology Oncology
16 - Home Ventilation 17 - ICU 18 - Immunology 19 - Infectious Diseases
20 - Internal Medicine 21 - Lung Medicine 22 - Metabolic Disorders 23 - Neonatal ICU
24 - Neonatology 25 - Nephrology 26 - Neurology 27 - Neurology Oncology
28 - Neurosurgery 29 - Neurosurgery Oncology 30 - Nuclear Medicine 31 - Oncology
32 - Ophthalmology 33 - Oral Surgery Oncology 34 - Oral Surgery 35 - Orthopedics
36 - Ped. Cardiothoracic Surgery 37 - Ped. Gastrointestinal and Liver Diseases
38 - Ped. Cardiology 39 - Ped. Endocrinology 40 - Ped. Hematology 41 - Ped. Immunology
42 - Ped. Lung Diseases 43 - Ped. Oncology 44 - Ped. Surgery 45 - Pediatrics
46 - Ped. ICU 47 - Plastic Surgery 48 - Radiology 49 - Radiotherapy
50 - Rheumatology 51 - Surgery 52 - Surgery Gastroenterology / Oncology
53 - Throat-Nose-Ear Medicine Oncology 54 - Throat-Nose-Ear Surgery 55 - Traumatology
56 - Urology 57 - Urology Oncology 58 - Vascular Medicine 59 - Vascular Surgery
