# Supplementary material for: Relevance of intra-hospital patient movements for the spread of healthcare-associated infections within hospitals - a mathematical modeling study
Source: PLoS Comput Biol. 2021 Feb 3;17(2):e1008600. doi: 10.1371/journal.pcbi.1008600 (PMC7857595; doi:10.1371/journal.pcbi.1008600)
Supplement: S4 Fig — (A) Complete CUM network without stratification, (B) Low-risk CUM network, (C) High-risk CUM network. Nodes represent departments and arrows represent patient movements between these departments. The color of the nodes was based on nodes degree whereas size of the nodes was based on nodes weighted degree. CBF, CCM and CVK are different campuses of the CUM hospital. (PDF) [file pcbi.1008600.s005.pdf]

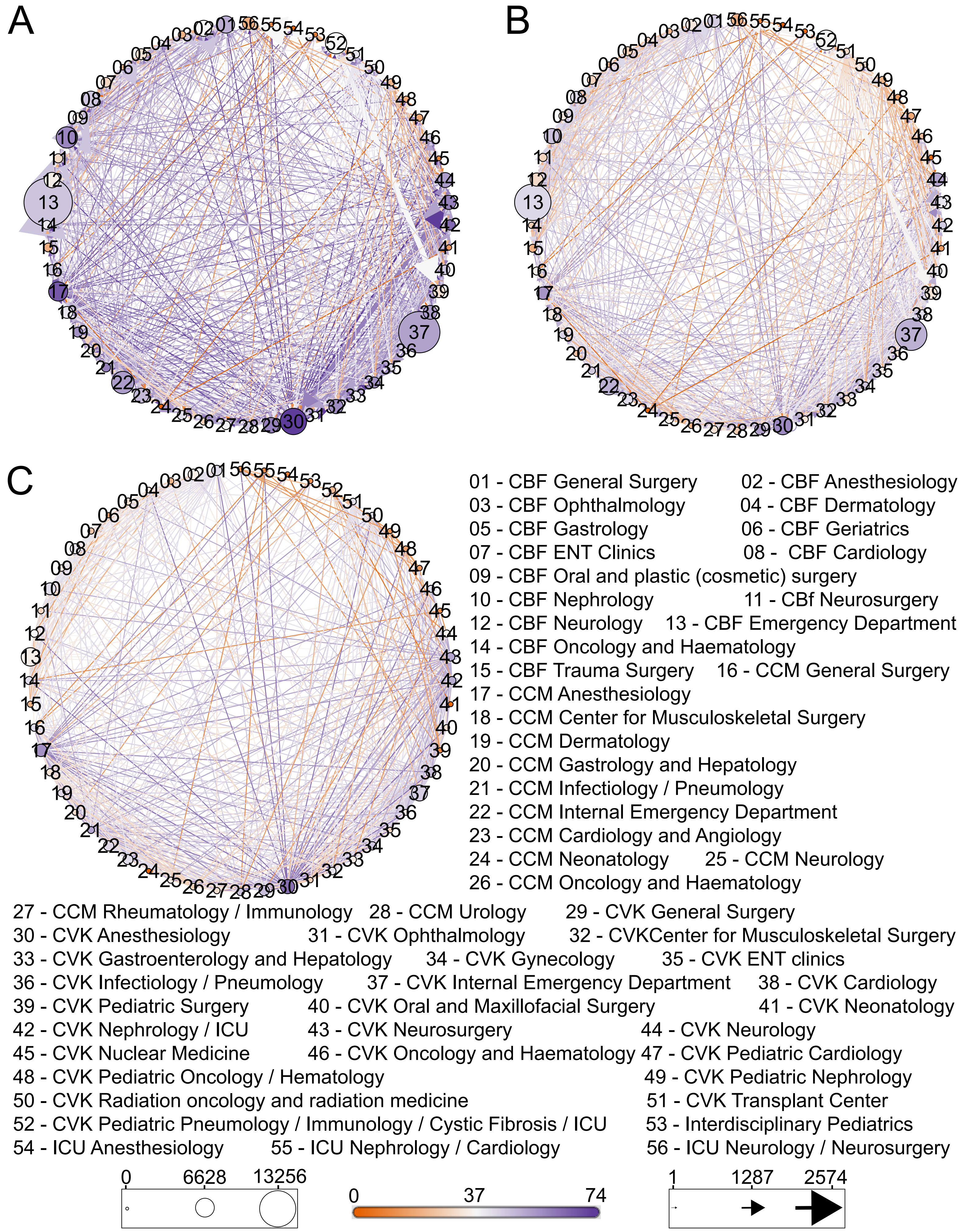
A
B
C
01 - CBF General Surgery
02 - CBF Anesthesiology
03 - CBF Ophthalmology
04 - CBF Dermatology
05 - CBF Gastrology
06 - CBF Geriatrics
07 - CBF ENT Clinics
08 - CBF Cardiology
09 - CBF Oral and plastic (cosmetic) surgery
10 - CBF Nephrology
11 - CBf Neurosurgery
12 - CBF Neurology
13 - CBF Emergency Department
14 - CBF Oncology and Haematology
15 - CBF Trauma Surgery
16 - CCM General Surgery
17 - CCM Anesthesiology
18 - CCM Center for Musculoskeletal Surgery
19 - CCM Dermatology
20 - CCM Gastrology and Hepatology
21 - CCM Infectiology / Pneumology
22 - CCM Internal Emergency Department
23 - CCM Cardiology and Angiology
24 - CCM Neonatology
25 - CCM Neurology
26 - CCM Oncology and Haematology
27 - CCM Rheumatology / Immunology
28 - CCM Urology
29 - CVK General Surgery
30 - CVK Anesthesiology
31 - CVK Ophthalmology
32 - CVKCenter for Musculoskeletal Surgery
33 - CVK Gastroenterology and Hepatology
34 - CVK Gynecology
35 - CVK ENT clinics
36 - CVK Infectiology / Pneumology
37 - CVK Internal Emergency Department
38 - CVK Cardiology
39 - CVK Pediatric Surgery
40 - CVK Oral and Maxillofacial Surgery
41 - CVK Neonatology
42 - CVK Nephrology / ICU
43 - CVK Neurosurgery
44 - CVK Neurology
45 - CVK Nuclear Medicine
46 - CVK Oncology and Haematology
47 - CVK Pediatric Cardiology
48 - CVK Pediatric Oncology / Hematology
49 - CVK Pediatric Nephrology
50 - CVK Radiation oncology and radiation medicine
51 - CVK Transplant Center
52 - CVK Pediatric Pneumology / Immunology / Cystic Fibrosis / ICU
53 - Interdisciplinary Pediatrics
54 - ICU Anesthesiology
55 - ICU Nephrology / Cardiology
56 - ICU Neurology / Neurosurgery
0
6628
13256
0
37
74
1
1287
2574
