# Supplementary figures and images for: Relevance of intra-hospital patient movements for the spread of healthcare-associated infections within hospitals - a mathematical modeling study
Source: PLoS Comput Biol. 2021 Feb 3;17(2):e1008600. doi: 10.1371/journal.pcbi.1008600 (PMC7857595; doi:10.1371/journal.pcbi.1008600)

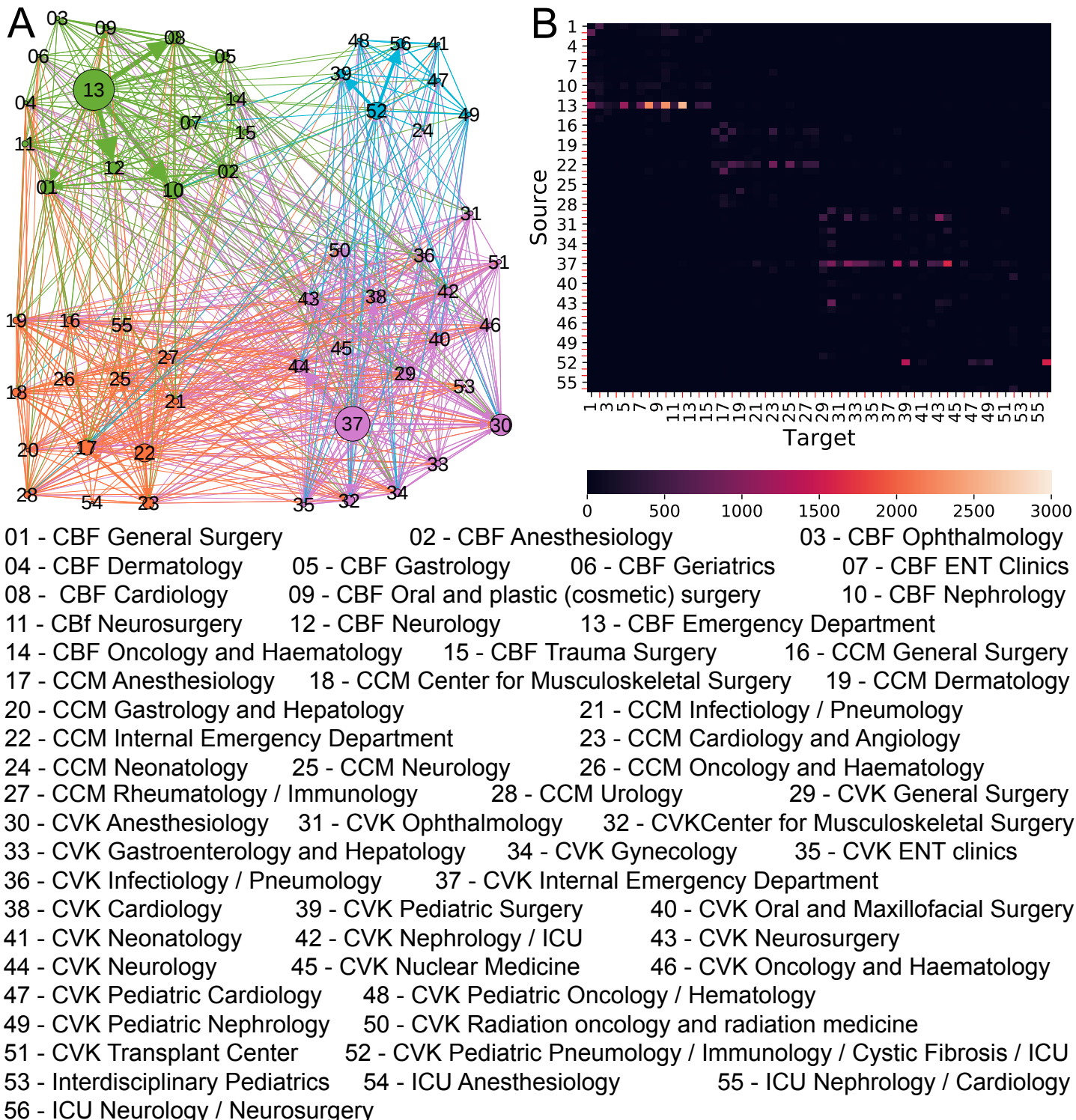

Supplement: S5 Fig — (A) Inter-department complete CUM hospital network showing clustering of the departments. Clustering is computed based on the modularity algorithm in the Gephi software which detects nodes that are more densely connected together than to the rest of the network. Node colors show the cluster to which a node belongs. The color of the arrow is based on the color of the node from where the arrow is originating. The thickness of the arrow is based on the number of patient’s transfers (weight). The size of the node is based on the weighted degree. (B) Heat map showing the number of transfers from one department to another department for the complete CUM network. A patient is transferred from the source to the target department. (PDF) [file pcbi.1008600.s006.pdf]

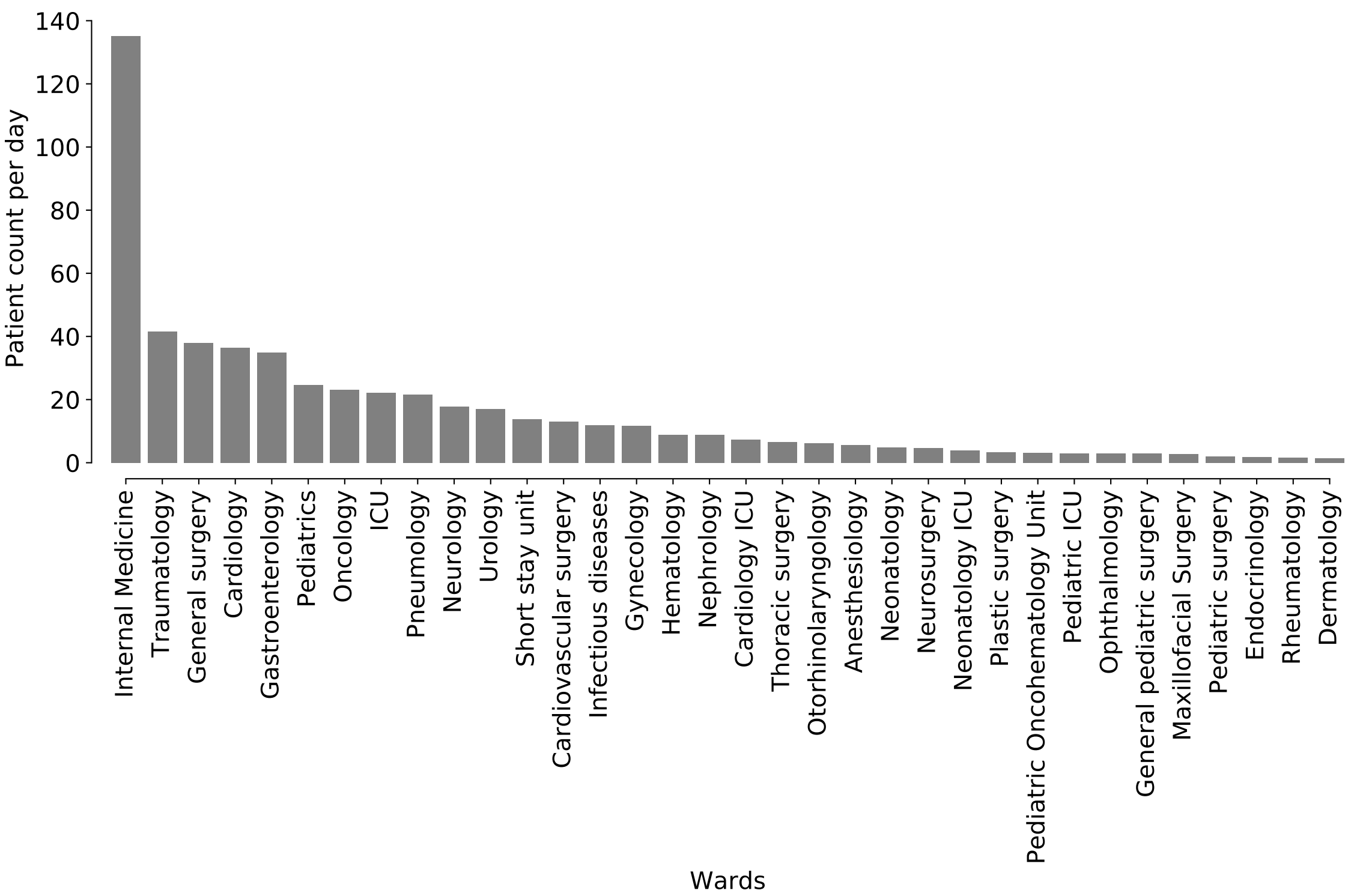

Supplement: S10 Fig — This data was used to define department size in terms of beds per department. (PDF) [file pcbi.1008600.s011.pdf]

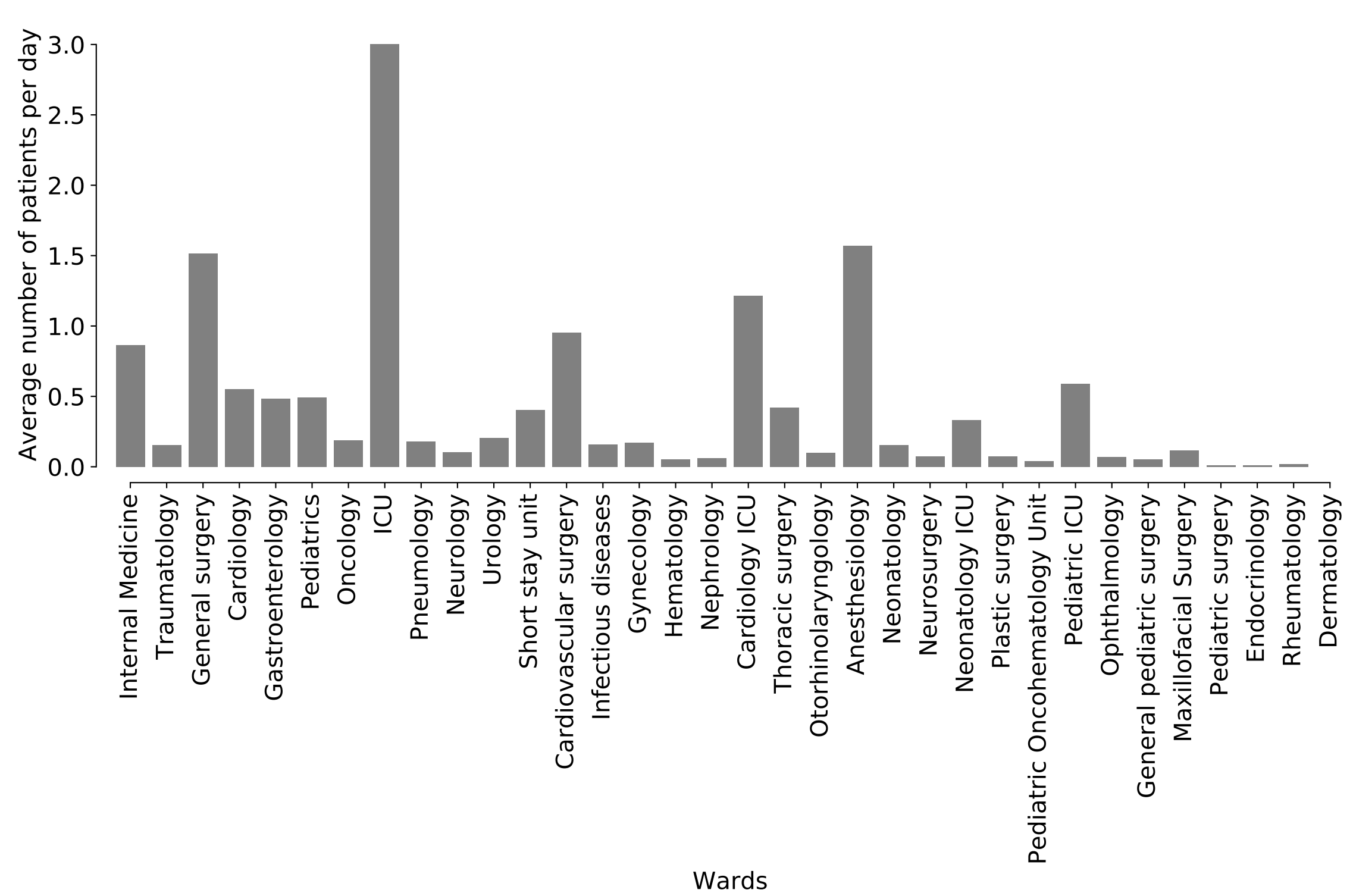

Supplement: S11 Fig — Departments are ordered by department size as shown in S10 Fig. (PDF) [file pcbi.1008600.s012.pdf]

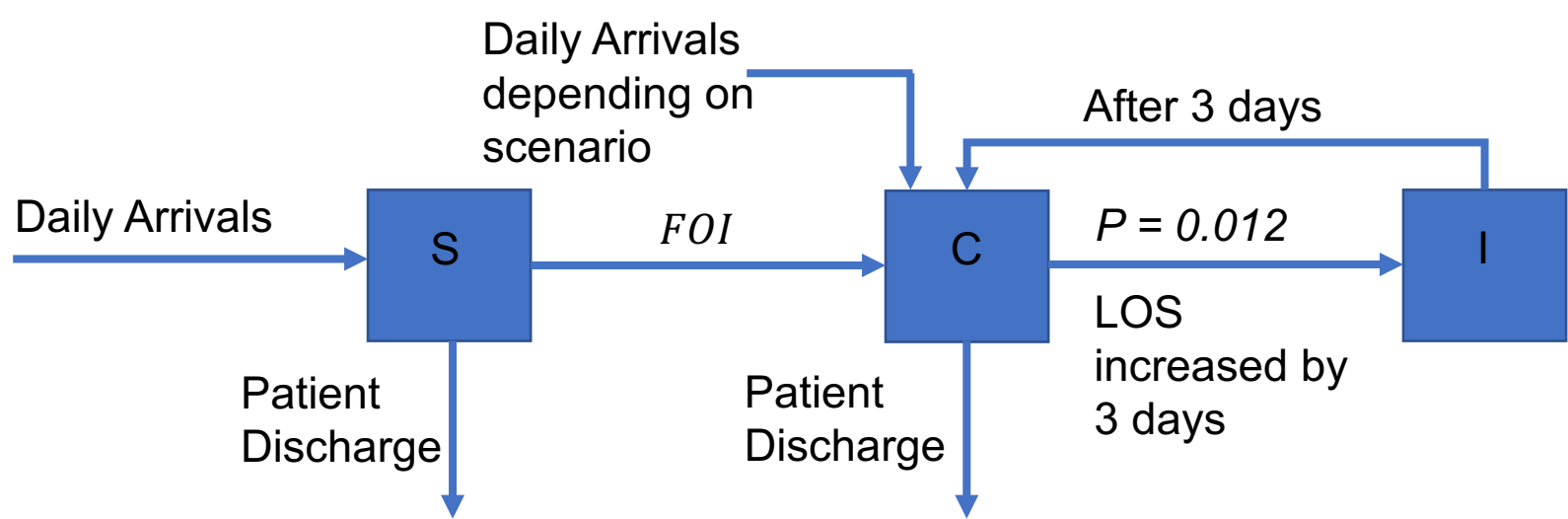

Supplement: S12 Fig — S refers to Susceptible, C refers to Colonized, and I refers to symptomatic infected patients. FOI is the force of infection. (PDF) [file pcbi.1008600.s013.pdf]

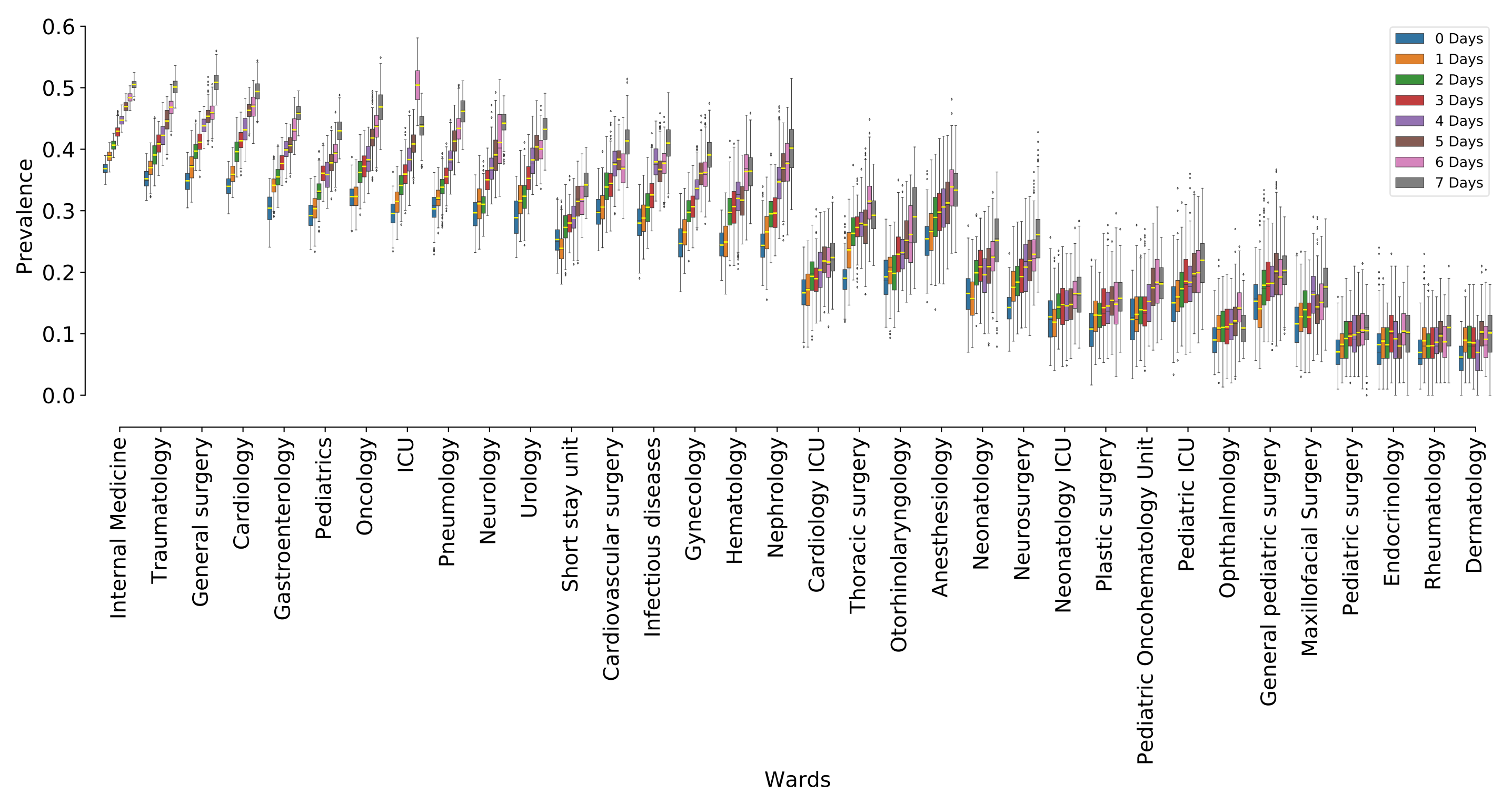

Supplement: S13 Fig — Transmission parameter β = 0.25 was used in each department. (PDF) [file pcbi.1008600.s014.pdf]

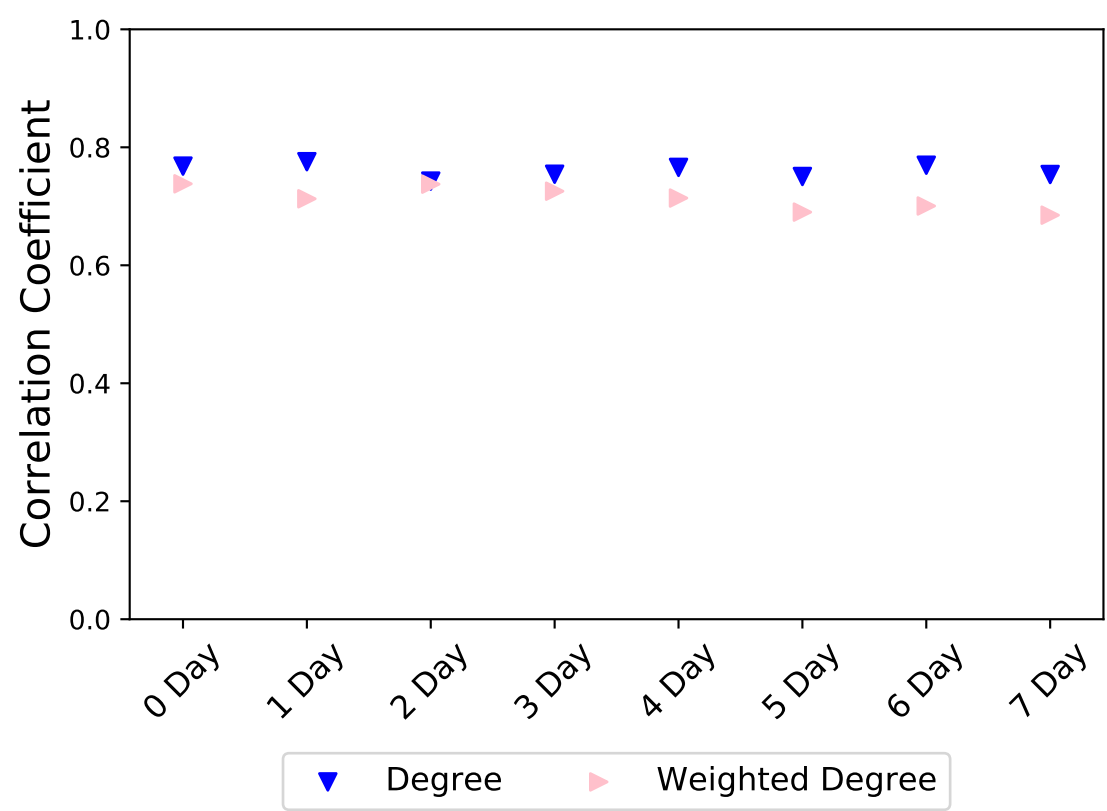

Supplement: S14 Fig — Different values for increase in the LOS for infected patients were used to identify the impact of this parameter on the correlation between steady state departments prevalence and network characteristics. Scenario 3 (5% continuous arrival of colonized patients) with transmission parameter β = 0.25 for every department was used. (PDF) [file pcbi.1008600.s015.pdf]
