# Supplementary material for: Relevance of intra-hospital patient movements for the spread of healthcare-associated infections within hospitals - a mathematical modeling study
Source: PLoS Comput Biol. 2021 Feb 3;17(2):e1008600. doi: 10.1371/journal.pcbi.1008600 (PMC7857595; doi:10.1371/journal.pcbi.1008600)
Supplement: S6 Fig — (A) Complete BH network without stratification, (B) Low-risk BH network, (C) High-risk BH network. Nodes represent departments and arrows represent patient movements between these departments. The color of the nodes was based on nodes degree whereas size of the nodes was based on the nodes’ weighted degree. (PDF) [file pcbi.1008600.s007.pdf]

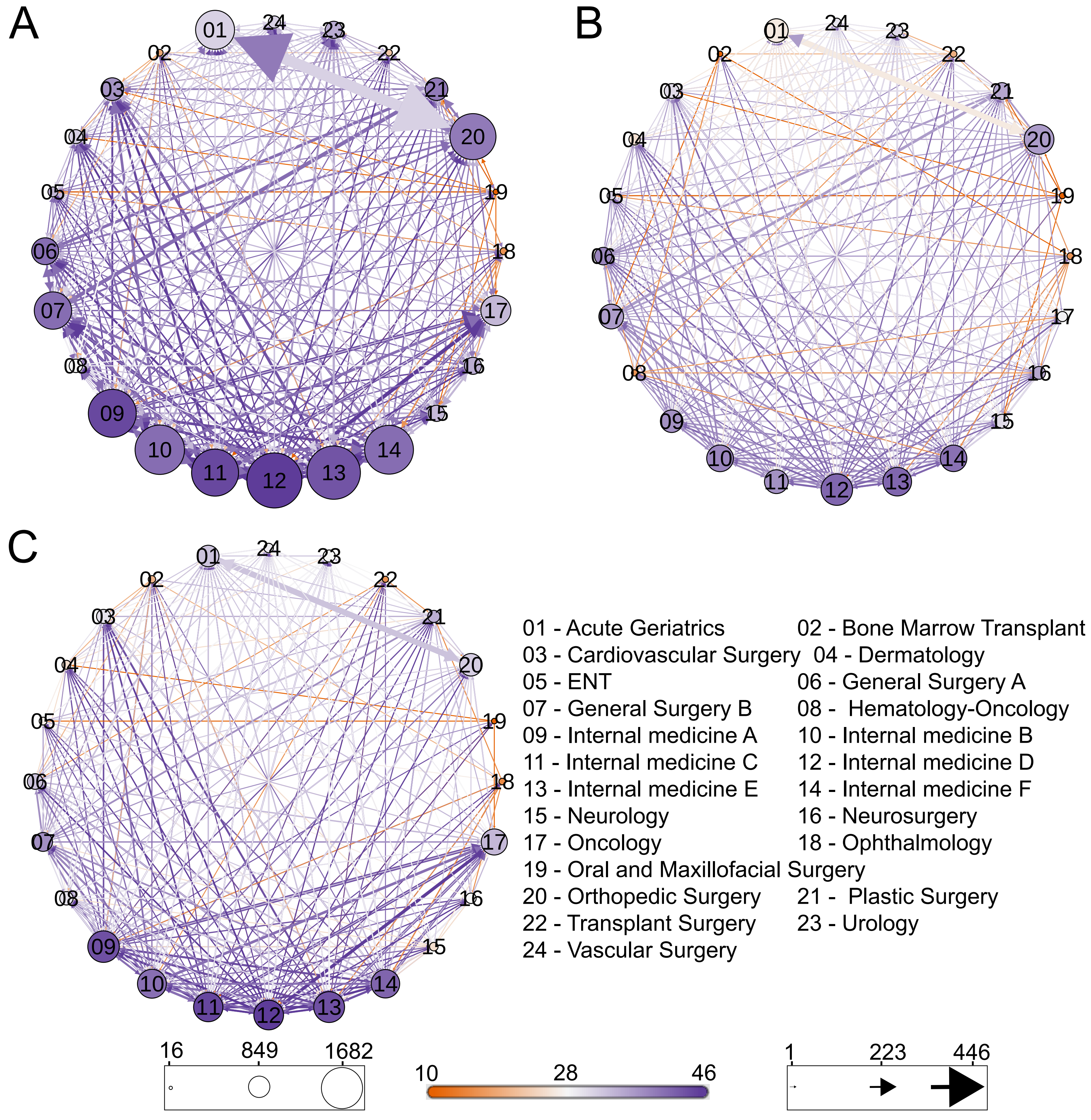
A
B
C
01 - Acute Geriatrics
02 - Bone Marrow Transplant
03 - Cardiovascular Surgery
04 - Dermatology
05 - ENT
06 - General Surgery A
07 - General Surgery B
08 - Hematology-Oncology
09 - Internal medicine A
10 - Internal medicine B
11 - Internal medicine C
12 - Internal medicine D
13 - Internal medicine E
14 - Internal medicine F
15 - Neurology
16 - Neurosurgery
17 - Oncology
18 - Ophthalmology
19 - Oral and Maxillofacial Surgery
20 - Orthopedic Surgery
21 - Plastic Surgery
22 - Transplant Surgery
23 - Urology
24 - Vascular Surgery
16
849
1682
10
28
46
1
223
446
