# Supplementary material for: Relevance of intra-hospital patient movements for the spread of healthcare-associated infections within hospitals - a mathematical modeling study
Source: PLoS Comput Biol. 2021 Feb 3;17(2):e1008600. doi: 10.1371/journal.pcbi.1008600 (PMC7857595; doi:10.1371/journal.pcbi.1008600)
Supplement: S7 Fig — (A) Inter-department complete BH hospital network showing clustering of the departments. Clustering is computed based on the modularity algorithm in the Gephi software which detects nodes that are more densely connected together than to the rest of the network. Node colors show the cluster to which a node belongs. The color of the arrow is based on the color of the node from where the arrow is originating. The thickness of the arrow is based on the number of patient’s transfers (weight). The size of the node is based on the weighted degree. (B) Heat map showing the number of transfers from one department to another department for the complete BH network. A patient is transferred from the source to the target department. (PDF) [file pcbi.1008600.s008.pdf]

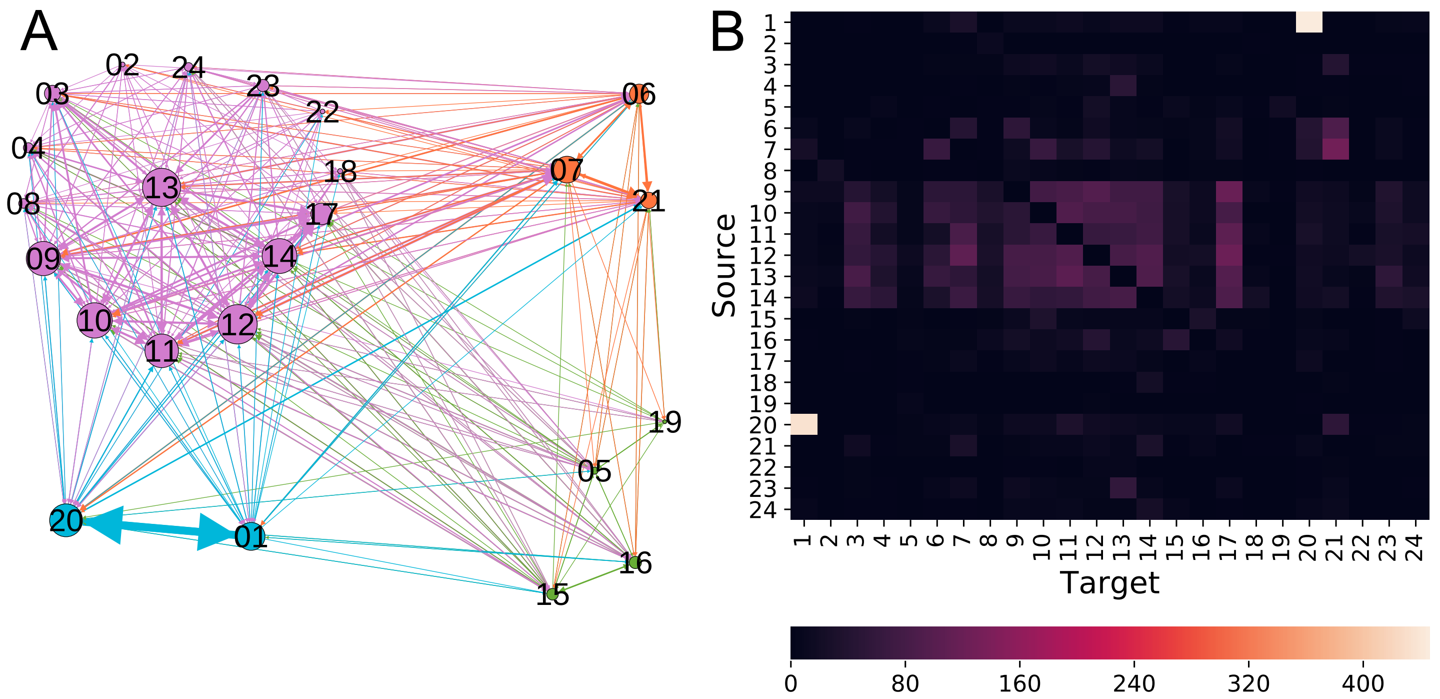

01 - Acute Geriatrics
02 - Bone Marrow Transplant
03 - Cardiovascular Surgery
04 - Dermatology
05 - ENT
06 - General Surgery A
07 - General Surgery B
08 - Hematology-Oncology
09 - Internal medicine A
10 - Internal medicine B
11 - Internal medicine C
12 - Internal medicine D
13 - Internal medicine E
14 - Internal medicine F
15 - Neurology
16 - Neurosurgery
17 - Oncology
18 - Ophthalmology
19 - Oral and Maxillofacial Surgery
20 - Orthopedic Surgery
21 - Plastic Surgery
22 - Transplant Surgery
23 - Urology
24 - Vascular Surgery
