# Supplementary material for: Relevance of intra-hospital patient movements for the spread of healthcare-associated infections within hospitals - a mathematical modeling study
Source: PLoS Comput Biol. 2021 Feb 3;17(2):e1008600. doi: 10.1371/journal.pcbi.1008600 (PMC7857595; doi:10.1371/journal.pcbi.1008600)
Supplement: S8 Fig — (A) Complete UKH network without stratification, (B) Low-risk UKH network, (C) High-risk UKH network. Nodes represent departments and arrows represent patient movements between these departments. The color of the nodes was based on nodes degree whereas size of the nodes was based on the nodes weighted degree. (PDF) [file pcbi.1008600.s009.pdf]

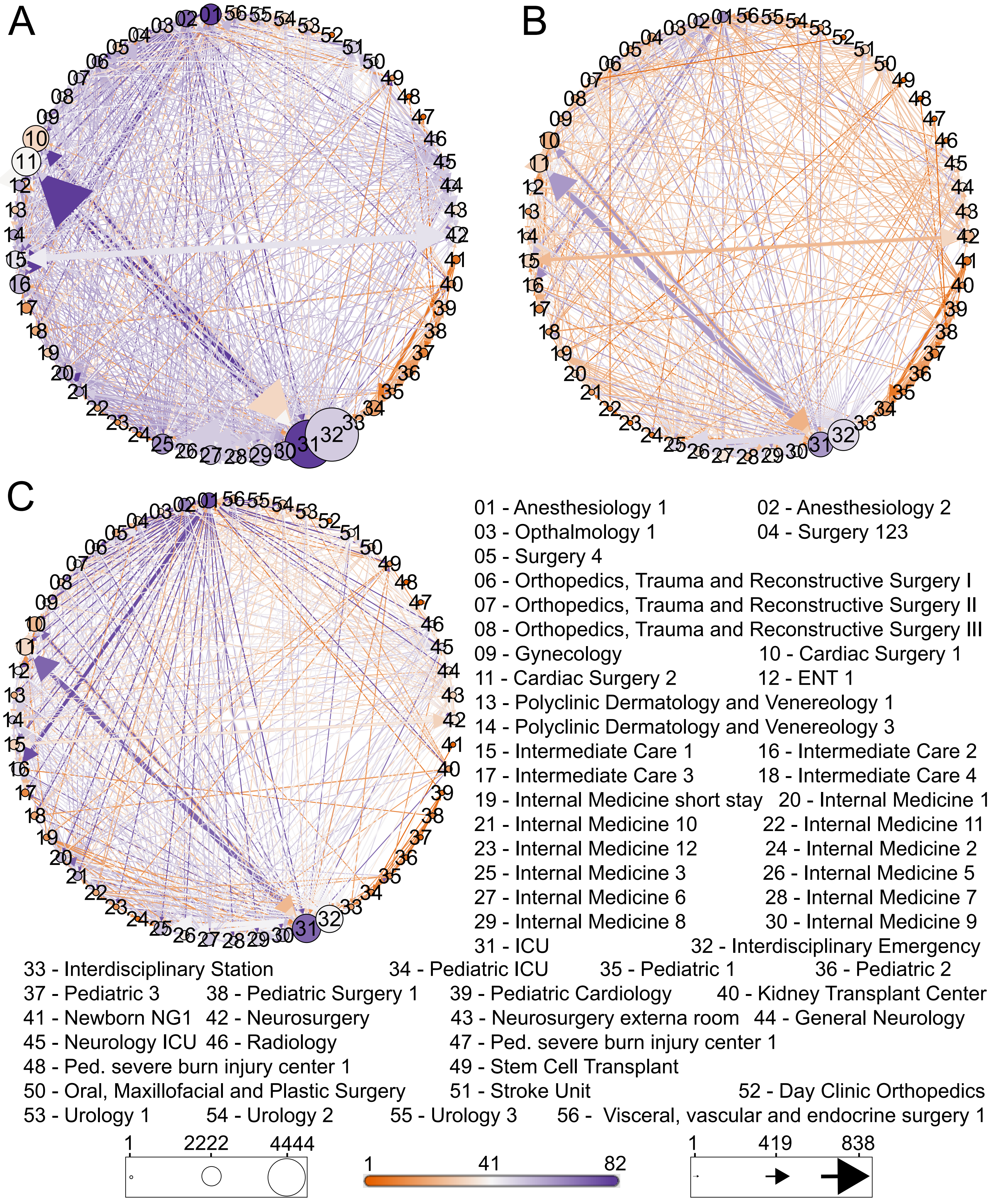
A
B
C
01 - Anesthesiology 1
02 - Anesthesiology 2
03 - Opthalmology 1
04 - Surgery 123
05 - Surgery 4
06 - Orthopedics, Trauma and Reconstructive Surgery I
07 - Orthopedics, Trauma and Reconstructive Surgery II
08 - Orthopedics, Trauma and Reconstructive Surgery III
09 - Gynecology
10 - Cardiac Surgery 1
11 - Cardiac Surgery 2
12 - ENT 1
13 - Polyclinic Dermatology and Venereology 1
14 - Polyclinic Dermatology and Venereology 3
15 - Intermediate Care 1
16 - Intermediate Care 2
17 - Intermediate Care 3
18 - Intermediate Care 4
19 - Internal Medicine short stay
20 - Internal Medicine 1
21 - Internal Medicine 10
22 - Internal Medicine 11
23 - Internal Medicine 12
24 - Internal Medicine 2
25 - Internal Medicine 3
26 - Internal Medicine 5
27 - Internal Medicine 6
28 - Internal Medicine 7
29 - Internal Medicine 8
30 - Internal Medicine 9
31 - ICU
32 - Interdisciplinary Emergency
33 - Interdisciplinary Station
34 - Pediatric ICU
35 - Pediatric 1
36 - Pediatric 2
37 - Pediatric 3
38 - Pediatric Surgery 1
39 - Pediatric Cardiology
40 - Kidney Transplant Center
41 - Newborn NG1
42 - Neurosurgery
43 - Neurosurgery externa room
44 - General Neurology
45 - Neurology ICU
46 - Radiology
47 - Ped. severe burn injury center 1
48 - Ped. severe burn injury center 1
49 - Stem Cell Transplant
50 - Oral, Maxillofacial and Plastic Surgery
51 - Stroke Unit
52 - Day Clinic Orthopedics
53 - Urology 1
54 - Urology 2
55 - Urology 3
56 - Visceral, vascular and endocrine surgery 1
1
2222
4444
1
41
82
1
419
838
