# Supplementary material for: Relevance of intra-hospital patient movements for the spread of healthcare-associated infections within hospitals - a mathematical modeling study
Source: PLoS Comput Biol. 2021 Feb 3;17(2):e1008600. doi: 10.1371/journal.pcbi.1008600 (PMC7857595; doi:10.1371/journal.pcbi.1008600)
Supplement: S9 Fig — (A) Inter-department complete UKH hospital network showing clustering of the departments. Clustering is computed based on the modularity algorithm in the Gephi software which detects nodes that are more densely connected together than to the rest of the network. Node colors show the cluster to which a node belongs. The color of the arrow is based on the color of the node from where the arrow is originating. The thickness of the arrow is based on the number of patient’s transfers (weight). The size of the node is based on the weighted degree. (B) Heat map showing the number of transfers from one department to another department for the complete UKH network. A patient is transferred from the source to the target department. (PDF) [file pcbi.1008600.s010.pdf]

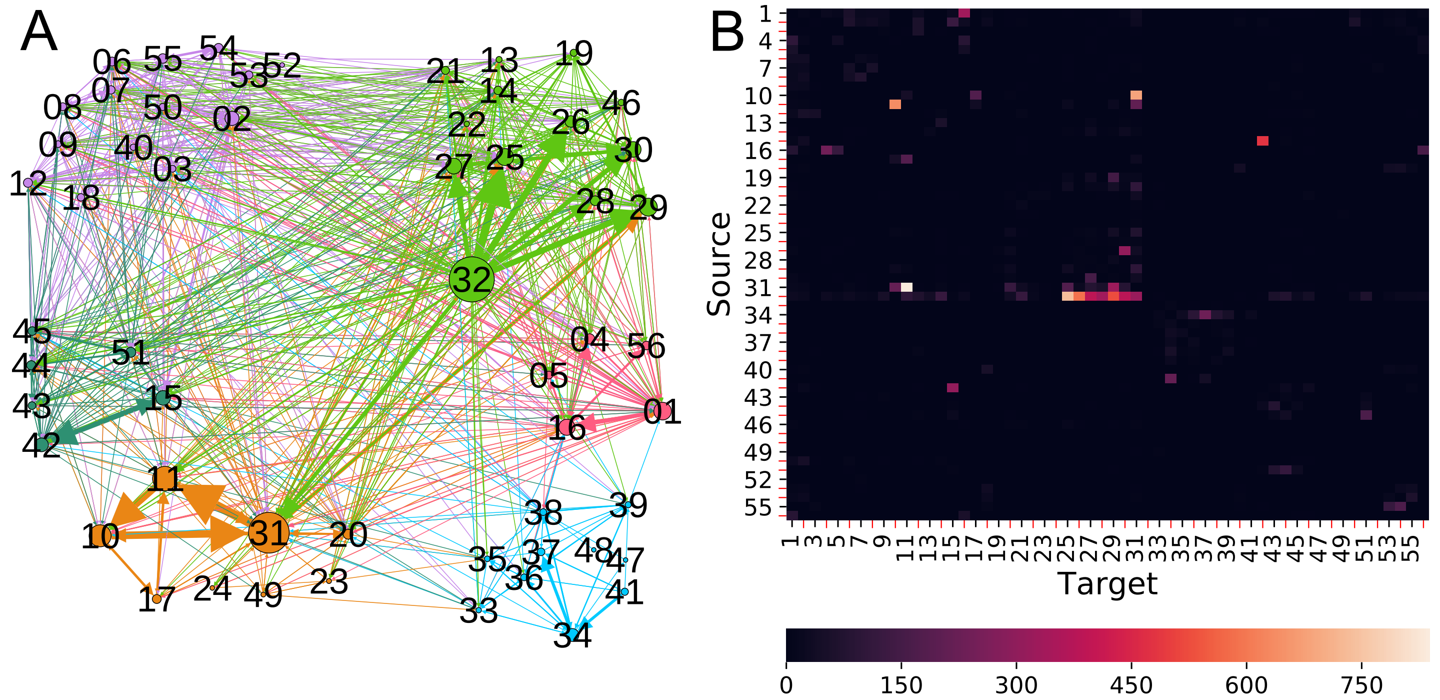

01 - Anesthesiology 1   02 - Anesthesiology   03 - Opthalmology 1   04 - Surgery 123
05 - Surgery 4   06 - Orthopedics, Trauma and Reconstructive Surgery I
07 - Orthopedics, Trauma and Reconstructive Surgery II   08 - Orthopedics, Trauma and Reconstructive Surgery III   09 - Gynecology   10 - Cardiac Surgery 1
11 - Cardiac Surgery 2   12 - ENT 1   13 - Polyclinic Dermatology and Venereology 1
14 - Polyclinic Dermatology and Venereology 3   15 - Intermediate Care 1
16 - Intermediate Care 2   17 - Intermediate Care 3   18 - Intermediate Care 4
19 - Internal Medicine short stay   20 - Internal Medicine 1   21 - Internal Medicine 10
22 - Internal Medicine 11   23 - Internal Medicine 12   24 - Internal Medicine 2
25 - Internal Medicine 3   26 - Internal Medicine 5   27 - Internal Medicine 6
28 - Internal Medicine 7   29 - Internal Medicine 8   30 - Internal Medicine 9
31 - ICU   32 - Interdisciplinary Emergency   33 - Interdisciplinary Station
34 - Pediatric ICU   35 - Pediatric 1   36 - Pediatric 2   37 - Pediatric 3
38 - Pediatric Surgery 1   39 - Pediatric Cardiology   40 - Kidney Transplant Center
41 - Newborn NG1   42 - Neurosurgery   43 - Neurosurgery externa room
44 - General Neurology   45 - Neurology ICU   46 - Radiology
47 - Ped. severe burn injury center 1   48 - Ped. severe burn injury center 1
49 - Stem Cell Transplant   50 - Oral, Maxillofacial and Plastic Surgery   51 - Stroke Unit
52 - Day Clinic Orthopedics   53 - Urology 1   54 - Urology 2   55 - Urology 3
56 - Visceral, vascular and endocrine surgery 1
