# Supplementary material for: Relevance of intra-hospital patient movements for the spread of healthcare-associated infections within hospitals - a mathematical modeling study
Source: PLoS Comput Biol. 2021 Feb 3;17(2):e1008600. doi: 10.1371/journal.pcbi.1008600 (PMC7857595; doi:10.1371/journal.pcbi.1008600)
Supplement: S15 Fig — Grey lines in the boxes show median of the data. (PDF) [file pcbi.1008600.s016.pdf]

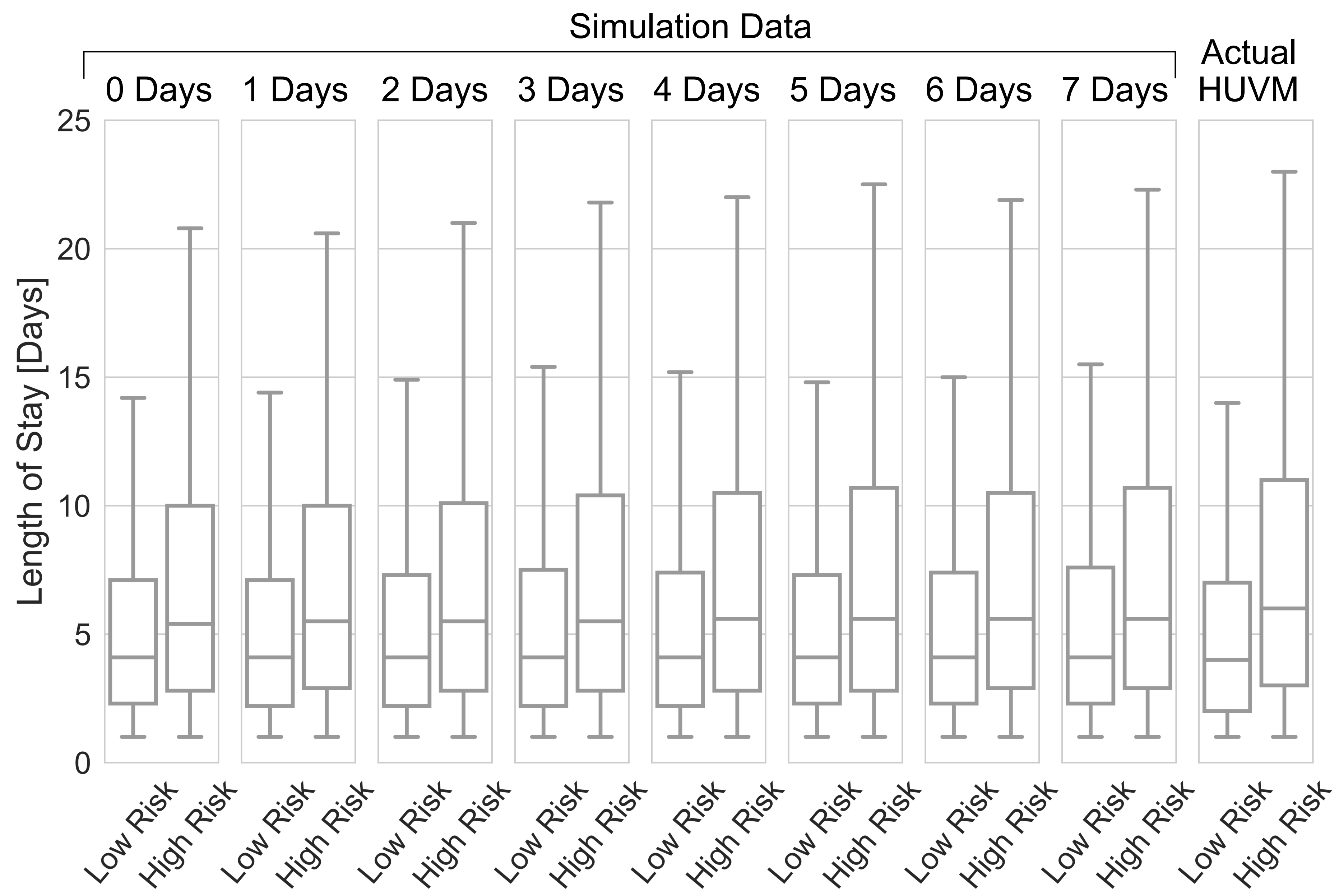
Simulation Data
0 Days
1 Days
2 Days
3 Days
4 Days
5 Days
6 Days
7 Days
Actual HUVM
Length of Stay [Days]
0
5
10
15
20
25
Low Risk
High Risk
